# From islet to blood: macrophage remodeling signatures for diagnosis and risk stratification in type 1 diabetes

**DOI:** 10.3389/fimmu.2026.1832672

**Published:** 2026-06-26

**Authors:** Yang Chen, Yiwen Zhou, Shuang Li, Yulu Chen, Jingfei Liu, Chun Yang, Hang Zhao, Zhangyao Su, Lingling Bian, Shuang Chen, Min Shen, Yao Qin, Heng Chen, Xinyu Xu, Yun Shi, Mei Zhang, Tao Yang, Yong Gu

**Affiliations:** Department of Endocrinology & Metabolism, The First Affiliated Hospital with Nanjing Medical University, Nanjing, Jiangsu, China

**Keywords:** biomarker, islet autoimmunity, machine learning, macrophage remodeling, peripheral blood (PB), risk stratification, single-cell RNA sequencing (scRNA seq), type 1 diabetes (T1D)

## Abstract

**Background:**

Type 1 diabetes (T1D) is an autoimmune disease characterized by progressive β-cell destruction, yet current risk stratification tools, which rely mainly on genetic susceptibility and autoantibody profiles, remain insufficient for accurately predicting disease progression. We aimed to characterize macrophage-related inflammatory transcriptional activity in T1D and to develop peripheral blood–based biomarkers for diagnosis and risk stratification.

**Methods:**

We integrated bulk RNA-seq, single-cell RNA-seq, and spatial transcriptomic data from human islets with public and in-house peripheral blood transcriptomic datasets. Macrophage heterogeneity and remodeling trajectories were analyzed in the islet microenvironment, and machine learning was used to derive tissue- and blood-based proinflammatory macrophage-related genes (PMRG). Diagnostic and prognostic models were then constructed and validated in peripheral blood cohorts, including a longitudinal islet autoimmunity (IA) cohort. SHAP analysis was applied to improve model interpretability. Independent PBMC RT-qPCR and mouse pancreatic immunofluorescence were performed to validate selected PMRG-related genes.

**Results:**

T1D islets showed marked immune remodeling with myeloid enrichment and five distinct macrophage subtypes. Pseudotime analysis identified a pro-inflammatory macrophage trajectory and 265 PMRGs, from which a 9-gene islet-derived PMRG (iPMRG) was obtained. Spatial transcriptomics supported the association of iPMRG-high macrophage signals with disrupted β-cell regions, and CellChat analysis inferred altered inflammatory communication programs. In peripheral blood mononuclear cells (PBMCs), the iPMRG-based diagnostic classifier distinguished T1D from healthy controls with an optimism-corrected AUC of 0.736. For prognosis, a 15-gene prognostic PMRGs was used to construct a risk score that, when integrated with clinical variables, predicted progression from IA to clinical T1D with time-dependent AUCs of 0.825, 0.814, and 0.860 at 12, 36, and 60 months, respectively. SHAP analysis identified the PMRG risk score as the dominant predictor and highlighted six core driver genes (PID1, TFPI2, SERPINB2, SOX4, DUSP2, and MT1X). The computational findings were further supported by independent validation in PBMCs and mouse pancreatic tissues.

**Conclusions:**

Our study highlights the heterogeneous and dynamic nature of macrophage remodeling in the T1D islet microenvironment, which is translated into accessible peripheral blood signatures. The resulting diagnostic and prognostic models provide an interpretable framework for T1D risk stratification and may support future strategies for earlier detection and precision prevention.

## Graphical Abstract

**Figure:**
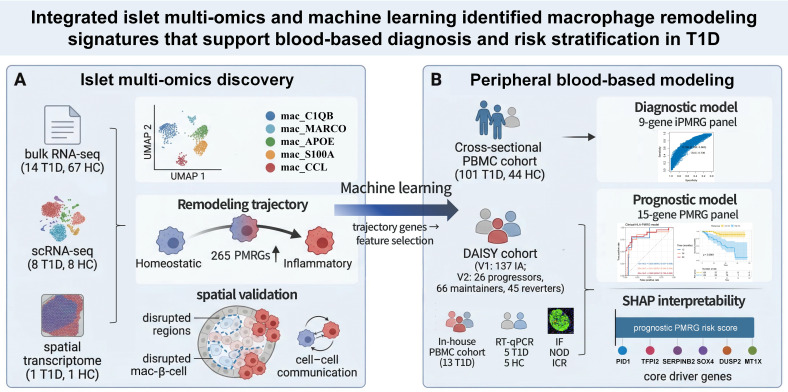

## Highlights

- We identified five macrophage subpopulations and a pro-inflammatory remodeling path in T1D islets, which shows clear macrophage diversity in the islet microenvironment.
- Spatial transcriptomics and cell–cell communication analysis provided exploratory support for inflammatory macrophage–β-cell microenvironmental interactions.
- Identified nine key iPMRGs and established a PBMC-based diagnostic model for T1D with an optimism-corrected AUC of 0.736.
- Developed a 15-gene peripheral blood prognostic model integrated with clinical features to predict progression from IA to T1D (AUCs = 0.825, 0.814, and 0.860 at 12, 36, and 60 months).
- SHAP analyses identified the PMRG risk score as the dominant predictor, and experimental validation provided additional support for selected key genes.

## 1 Introduction

Type 1 diabetes (T1D) is a chronic autoimmune disease characterized by progressive destruction of pancreatic β-cells, resulting in absolute insulin deficiency and hyperglycemia (1). Despite advances in insulin therapy, T1D remains a major global health challenge due to its increasing incidence and the lifelong burden of disease management (2). Current risk assessment still relies largely on genetic risk scores, islet autoantibodies and related clinical features (3–5), yet these approaches remain insufficient for precisely identifying which individuals with islet autoimmunity (IA) will progress to overt clinical T1D. Therefore, there is a pressing need for biomarkers that better reflect disease-relevant immune activity and improve early risk stratification (6, 7).

The pathogenesis of T1D involves a complex interplay among genetic predisposition, environmental triggers, and immune-mediated β-cell destruction (3). Although autoreactive T cells have been extensively studied in T1D (8), the contribution of innate immune cells, particularly macrophages, has attracted increasing attention in recent years (9). Macrophages are highly plastic immune cells that play critical roles in tissue homeostasis, inflammation, and host defense (10). In response to local microenvironmental cues, they can adopt distinct functional states, including pro-inflammatory and tissue-repair–associated programs (11, 12). In autoimmune diseases, dysregulated macrophage polarization has been implicated in disease initiation, amplification of local inflammation, and tissue injury (13, 14). In T1D, macrophages have been linked to antigen presentation, cytokine production, and modulation of T-cell responses, suggesting that they may participate in both the establishment and persistence of islet inflammation (15–17).

However, several important questions remain unresolved. Although the classical M1/M2 model provides a useful framework for describing broad inflammatory and reparative states, it does not fully capture the functional diversity of macrophages in complex tissues (18). Instead, macrophage activation is increasingly understood as a dynamic, tissue-shaped program influenced by local immune context, metabolic or stress-related cues, and cell–cell interactions (19). In this setting, a comprehensive understanding of macrophage heterogeneity, dynamic remodeling within the T1D islet microenvironment is still lacking. In particular, the molecular signatures associated with disease-enriched inflammatory macrophage states, and their detectability in peripheral blood have not been systematically characterized. Advances in single-cell RNA sequencing (scRNA-seq) have greatly improved the ability to resolve cellular heterogeneity and functional states at high resolution (20), while integration with machine learning provides an opportunity to extract biologically meaningful signatures with potential clinical utility (21).

In this study, we integrated islet bulk RNA-seq, scRNA-seq, spatial transcriptomic, and peripheral blood transcriptomic datasets to characterize macrophage-related inflammatory transcriptional activity in T1D. We aimed to define macrophage subpopulations and remodeling trajectories within the islet microenvironment, identify core proinflammatory macrophage signatures, and evaluate whether these tissue-derived signals could be translated into accessible peripheral blood biomarkers. On this basis, we developed and validated a peripheral blood-derived diagnostic model for T1D and a prognostic model for predicting progression from IA to clinical T1D, while further enhancing model interpretability through SHAP-based analyses.

## 2 Results

### 2.1 Integrated bulk and single-cell transcriptomics reveal immune activation and myeloid enrichment in T1D islets

**Figure 1:**
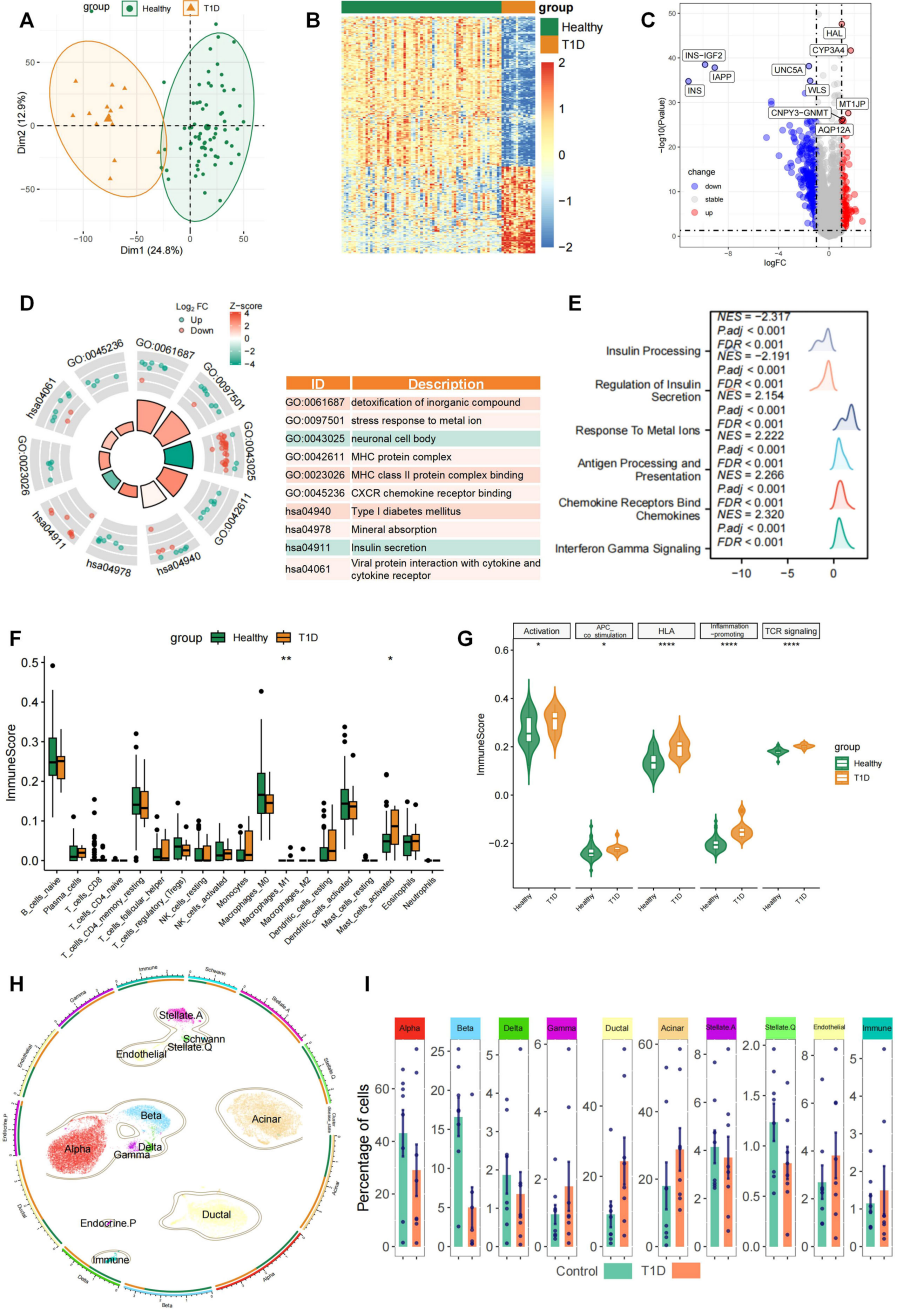
Integrated bulk and single-cell transcriptomics reveal immune activation and myeloid dominance in T1D islets. **(A)** performed principal component analysis (PCA) plot of transcriptomic features of type 1 diabetes (T1D) and healthy controls. **(B)** Heatmap and **(C)** volcano plot showing 378 differentially expressed genes (DEGs) between T1D and healthy islet samples, including 138 upregulated genes (shown in red) and 240 downregulated genes (shown in blue). Thresholds were adjusted P-value < 0.05 and |logFC| > 1. **(D)** Gene Ontology (GO) and Kyoto Encyclopedia of Genes and Genomes (KEGG) pathway enrichment of DEGs. **(E)** Gene Set Enrichment Analysis results of DEGs. Thresholds were adjusted P-value < 0.05, |NES| > 1, and FDR q-value < 0.25. **(F)** Immune infiltrating cell components in T1D and healthy islet tissues analyzed by CIBERSORT. **(G)** Immune pathway and function scores of T1D and healthy islet tissues analyzed by single-sample Gene Set Enrichment Analysis (ssGSEA). **(H)** Cell types were identified by comparing classical marker genes and cluster marker genes. A total of 12 major cell types were identified, including α cells (Alpha), β cells (Beta), δ cells (Delta), γ cells (Gamma), proliferating endocrine cells (Endo.P), acinar cells, quiescent stellate cells (Stellate.Q), activated stellate cells (Stellate.A), endothelial cells, ductal cells, Schwann cells, and immune cells. **(I)** Proportions of different cell types in the islet microenvironment of type 1 diabetes (T1D) and healthy controls. * indicates P < 0.05, ** indicates P < 0.01, *** indicates P < 0.001, **** indicates P < 0.0001.

To assess the biological functions involved in the onset and progression of type 1 diabetes (T1D), we utilized transcriptome datasets from public databases (22–24), focusing on islet samples (GSE181674 (22), GSE50244 (23), GSE162689 (24)). We conducted a comprehensive evaluation of the expression patterns between 14 T1D patients and 67 healthy controls (HC). The results revealed significant differences in the transcriptomic features of islet tissues between T1D patients and healthy controls (**Figures 1A, B**). There were 378 differentially expressed genes (DEGs) between the two groups (|logFC| > 1 and adjusted P-value < 0.05), including 138 upregulated genes and 240 downregulated genes (**Figure 1C**). Functional annotation indicated that these DEGs were mainly enriched in immune activation pathways, including cytokine-cytokine receptor interaction, antigen processing and presentation, interferon-γ signaling, and chemokine-mediated pathways, while pathways related to insulin processing and secretion were significantly reduced (**Figures 1D, E**; **Supplementary Tables 1-2**). Consistently, immune deconvolution using CIBERSORT (25) revealed higher proportions of M1 macrophages and activated mast cells in T1D islets (**Figure 1F**), and single-sample Gene Set Enrichment Analysis (ssGSEA) (26–28) also showed higher scores for inflammation-promoting, HLA, and antigen-presenting cell co-stimulatory, which supports enhanced immune activation and TCR signaling in T1D (**Figure 1G**).

To further elucidate the immune landscape, we constructed an atlas using single-cell RNA sequencing (scRNA-seq) data from eight T1D and eight healthy control islet samples, yielding 30,497 high-quality cells after quality control and data integration (**Supplementary Figures 1A–C**). A total of 12 major cell types were identified, including α cells, β cells, δ cells, γ cells, proliferating endocrine cells, acinar cells, stellate cells (in both quiescent and activated states), endothelial cells, ductal cells, Schwann cells, and immune cells (**Figure 1H**). The marker genes of each cell type were illustrated using bubble plots (**Supplementary Figure 1D**). Importantly, comparative compositional analysis demonstrated a significant reduction of α and β cells alongside an expansion of γ cells, ductal cells, acinar cells, endothelial cells, and immune cells in T1D islets (**Figure 1I**), jointly indicating that myeloid-dominant immune infiltration is a prominent feature of the T1D islet and likely contributes to local inflammatory remodeling and endocrine dysfunction.

### 2.2 Single-cell transcriptomic atlas reveals five distinct macrophage subgroups in T1D islets

**Figure 2:**
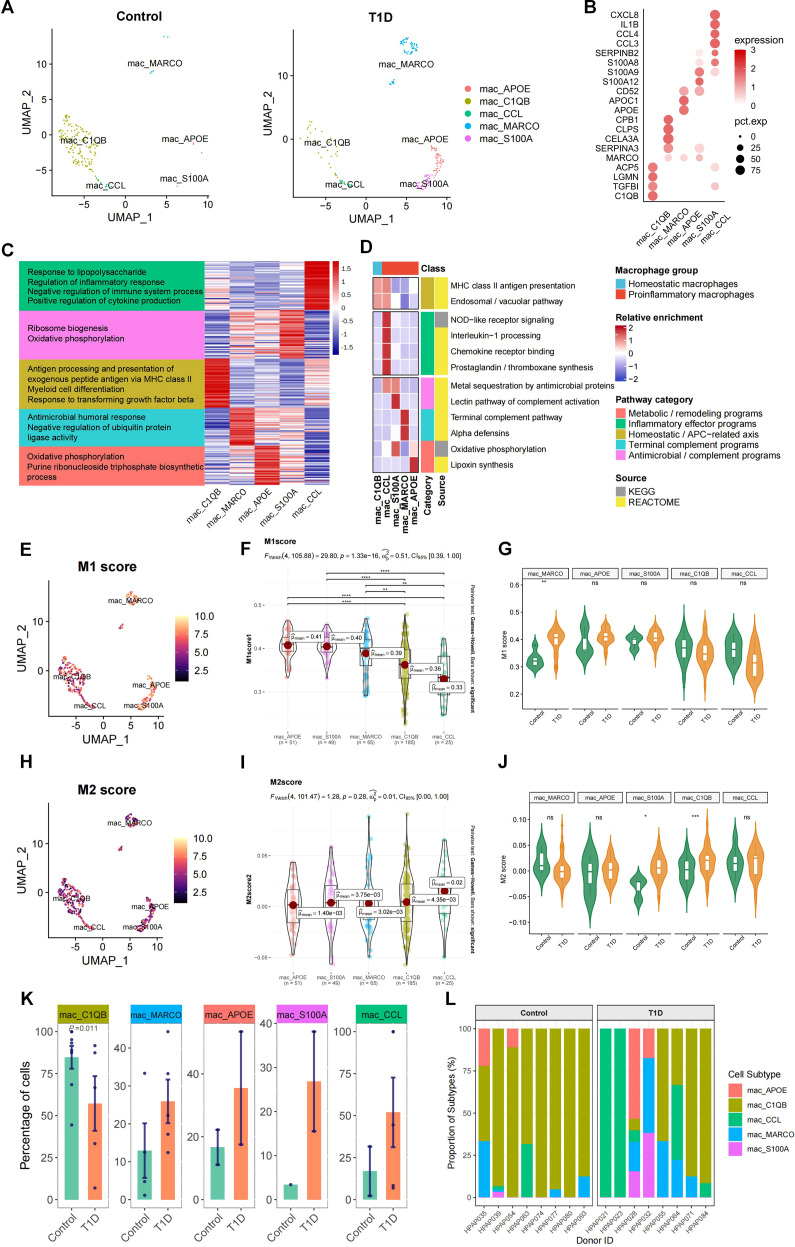
Identification of islet-infiltrating immune cells in type 1 diabetes and healthy controls. **(A)** UMAP dimensionality reduction clustering of islet-infiltrating macrophage subtypes in type 1 diabetes (T1D) and healthy controls. Macrophages were identified by comparing classical markers and cluster marker genes, resulting in the identification of five major subtypes: mac_APOE, mac_S100A, mac_MARCO, mac_C1QB and mac_CCL. **(B)** Bubble plot illustrating the marker genes for each subgroup. **(C)** Differentially expressed genes among cell subgroups were identified using the FindAllMarkers function and visualized with heatmaps. **(D)** Curated GSVA heatmap of representative KEGG and REACTOME pathways across the five macrophage subgroups. **(E)** Featureplot depicting M1 scores of islet-infiltrating macrophages in T1D and healthy controls. **(F)** M1 score differences among macrophage subgroups. **(G)** M1 score differences between macrophage subgroups in T1D and healthy controls. **(H)** FeaturePlot depicting M2 scores of islet-infiltrating macrophages in T1D and healthy controls. **(I)** M2 score differences among macrophage subgroups. **(J)** M2 score differences between macrophage subgroups in T1D and healthy controls. **(K)** Proportions of macrophage subgroups infiltrating the islets in T1D and healthy controls. **(L)** Donor-level proportions of five macrophage subgroups in control and T1D samples. * indicates P < 0.05, ** indicates P < 0.01, *** indicates P < 0.001, **** indicates P < 0.0001.

Given the significant increase in immune cells, especially M1 macrophages, within the islets of type 1 diabetes (T1D) patients, we selected macrophages expressing C1QC, C1QA, CD68 for further dimensionality reduction and clustering analysis. Utilizing cell type-specific marker genes reported in the literature (29–31), we identified five major macrophage subtypes, including mac_APOE, mac_S100A, mac_MARCO, mac_CCL, and mac_C1QB (**Figure 2A**). The marker genes of each cell subtype were visualized in a bubble plot (**Figure 2B**).

To elucidate the functional characteristics of the five macrophage subgroups, we conducted GO enrichment analysis on their respective DEGs (**Figure 2C**; **Supplementary Table 3**). The mac_APOE, mac_S100A and mac_MARCO subgroups were mainly enriched in biosynthetic and metabolic pathways, including cytoplasmic translation and ribonucleoprotein complex biogenesis. The mac_APOE and mac_S100A additionally showed oxidative phosphorylation and purine ribonucleoside triphosphate biosynthetic features, whereas mac_MARCO was uniquely related to antimicrobial humoral response. In contrast, mac_CCL and mac_C1QB were both linked to exogenous peptide antigen processing and presentation. Despite this commonality, they displayed divergent functional profiles: the mac_CCL subgroup was involved in response to lipopolysaccharide and cytokine production, whereas mac_C1QB was associated with myeloid cell differentiation and response to transforming growth factor beta. Curated Gene Set Variation Analysis (GSVA) using KEGG and REACTOME gene sets further supported the functional divergence among the five subgroups (**Figure 2D**). Meanwhile, based on the M1 and M2 characteristic gene sets reported in the literature (32), we employed the AddModuleScore function to evaluate polarization-related signatures across macrophage subgroups. The mac_APOE, mac_S100A and mac_MARCO subgroups exhibited the highest M1 scores, followed by mac_C1QB subgroup, while the mac_CCL subgroup displayed the lowest M1 score (**Figures 2E, F**). Notably, the M1 score of mac_MARCO was higher in T1D than in healthy controls (**Figure 2G**). In contrast, overall M2 scores did not differ significantly across subgroups (**Figures 2H, I**), although mac_S100A and mac_C1QB showed higher M2 scores in T1D than in healthy controls (**Figure 2J**). Together, these findings support functional heterogeneity across the five macrophage subgroups within the T1D islet microenvironment.

Consistent with their distinct functional annotations, the five macrophage subgroups also differed in composition between control and T1D islets. T1D islets were predominantly composed of mac_APOE, mac_S100A, mac_MARCO, and mac_CCL, whereas control islets were mainly enriched in mac_C1QB (**Figure 2K**). Accordingly, mac_APOE, mac_S100A, mac_MARCO, and mac_CCL were operationally grouped as proinflammatory macrophages for downstream analyses, whereas mac_C1QB was used as the homeostatic macrophage subgroup. Donor-level analysis confirmed the same overall shift and showed that it was not driven by any individual donor (**Figure 2L**; **Supplementary Figures 2A–D**).

### 2.3 Pseudotime trajectory analysis suggests a pro-inflammatory differentiation path for islet macrophages

**Figure 3:**
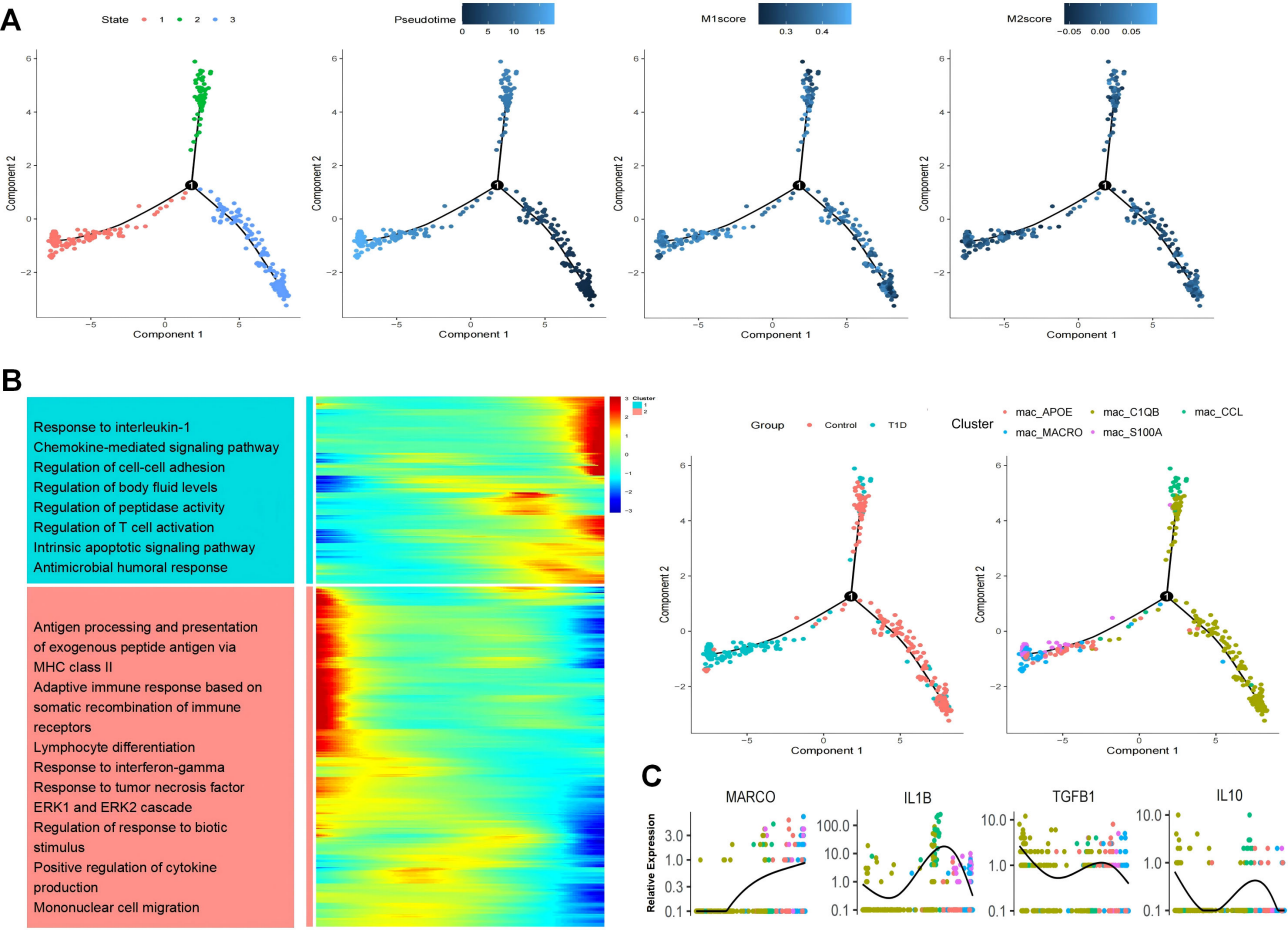
Pseudotime analysis of islet-infiltrating macrophages in type 1 diabetes and healthy controls. **(A)** The Monocle2 algorithm employs machine learning to order the macrophage differentiation process and construct trajectories. Each state represents distinct movement phases of the cells. Pseudotime is a time probability calculated based on cellular gene expression data, indicating the sequence of differentiation. The group represents disease classification, while the cluster denotes macrophage subgroups. M1 score indicates the M1 polarization score of each cell. M2 score indicates the M2 polarization score of each cell. **(B)** Differentially expressed genes (DEGs) along the macrophage pseudotime trajectory were clustered into two modules based on kinetic trends, and Gene Ontology (GO) functional enrichment analyses were performed for the DEGs in each module. **(C)** Changes in the expression of marker genes for M1 macrophages (MARCO, IL1B) and M2 macrophages (TGFB1, IL10) along pseudotime.

Using the Monocle2 algorithm (33), we conducted pseudotime trajectory analysis to infer transcriptional changes associated with macrophage remodeling in T1D. As shown in **Figure 3A**, pseudotime analysis suggested an ordered trajectory from mac_C1QB toward multiple T1D-enriched macrophage states, which could be divided into three distinct states. In healthy individuals, macrophages were predominantly of the mac_C1QB subgroup, mainly located in the early pseudotime states. Consistent with the subgroup-level functional classification, the late trajectory states were dominated by the four T1D-enriched proinflammatory macrophage subgroups. Visualization of the M1 scores of macrophages along the pseudotime trajectory indicates that canonical M1-associated signatures were more prominent in the late pseudotime states.

We further analyzed the molecular regulatory features underlying this pathogenic transition (**Supplementary Table 4**). In **Figure 3B**, genes exhibiting similar kinetic trends during macrophage differentiation were clustered into two modules. Module 1 includes 265 pseudotime DEGs, including MARCO and IL1B, whose expression levels increase over time. Module 2 contains 482 pseudotime DEGs, including TGFB1 and IL10, whose expression levels decrease over time (**Figure 3C**). GO enrichment analysis revealed two contrasting biological programs. Module 1 was associated with inflammatory sensing and leukocyte recruitment (response to interleukin-1, chemokine-mediated signaling, regulation of cell–cell adhesion), immune activation (regulation of peptidase activity, regulation of T-cell activation), and effector responses (antimicrobial humoral response, intrinsic apoptotic signaling). In contrast, Module 2 captured a coordinated decline in homeostatic immune surveillance functions, particularly antigen-presenting cells (APCs) and cytokine responsive (response to interferon-γ and tumor necrosis factor). Collectively, these results suggest that macrophage progression along pseudotime is accompanied by activation of proinflammatory effector programs (Module 1) alongside suppression of immune surveillance and cytokine-responsive homeostatic functions (Module 2) (**Figure 3B**). Accordingly, the Module 1 gene set was designated as proinflammatory macrophage-related genes (PMRGs) for downstream analyses.

### 2.4 iPMRGs are functionally linked to β cell interaction and *in-situ* damage

**Figure 4:**
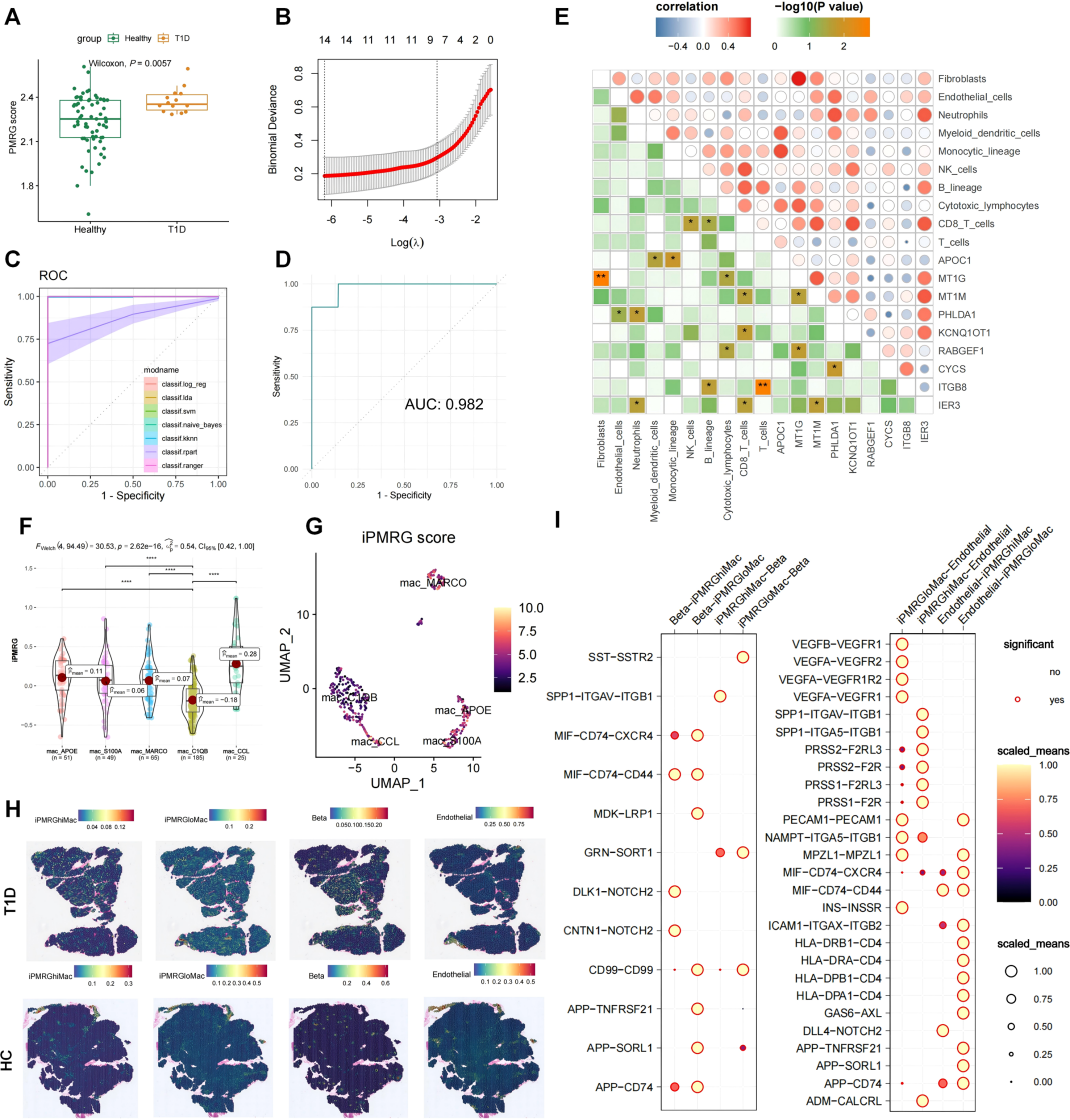
Identification of key genes for proinflammatory macrophage remodeling in T1D using machine learning. **(A)** Gene Set Variation Analysis (GSVA) scoring of proinflammatory macrophage-related genes (PMRG) in the islet transcriptome of type 1 diabetes (T1D) compared to healthy individuals. **(B)** Transcriptomic data from T1D patients and healthy individuals were divided into training and validation sets, with Least Absolute Shrinkage and Selection Operator (LASSO) regression employed to select genes for model construction in the training set, ultimately identifying nine key genes associated with proinflammatory macrophage remodeling. **(C)** Comparison of the area under the curve (AUC) of models constructed using seven machine learning algorithms, resulting in the selection of the Naive Bayes model based on these findings. **(D)** The AUC of receiver operating characteristic (ROC) analysis of the validation set using the Naive Bayes model. **(E)** Heatmap illustrating the correlation between the nine key islet-derived PMRG (iPMRGs) and immune cell infiltration scores. **(F)** Violin plots comparing iPMRG scores across macrophage subgroups. **(G)** UMAP showing iPMRG score distribution. **(H)** Spatial feature plots of iPMRGhiMac, iPMRGloMac, β-cell and endothelial markers in one T1D sample and one healthy control sample. **(I)** CellChat-inferred differential communication programs of iPMRGhiMac versus iPMRGloMac. * indicates P < 0.05, ** indicates P < 0.01, *** indicates P < 0.001, **** indicates P < 0.0001.

GSVA scoring (34) was performed on islet transcriptomic data using 265 PMRG, revealing that the PMRG score in T1D patients were significantly higher than those in healthy individuals (**Figure 4A**). Subsequently, datasets GSE181674 (22) and GSE50244 (23) were utilized as the training set, while GSE162689 (24) served as the validation set for conducting univariate logistic regression (**Supplementary Table 5**) and Least Absolute Shrinkage and Selection Operator (LASSO) regression on PMRG (**Figure 4B**). A total of nine key islet-derived PMRG (iPMRG) with diagnostic potential for T1D were identified (APOC1, MT1G, MT1M, PHLDA1, KCNQ1OT1, RABGEF1, CYCS, ITGB8, IER3). We then applied seven machine learning algorithms and optimized each model using five repeated ten-fold cross-validation (**Figure 4C**). After comprehensively evaluating the model performance, the Naive Bayes model was ultimately selected, achieving an area under the curve (AUC) of 0.982 in the receiver operating characteristic (ROC) analysis of the validation set (**Figure 4D**). Additionally, we evaluated the association of the nine iPMRGs with islet immune infiltrating patterns (**Figure 4E**). Notably, APOC1 showed a significant positive correlations with monocyte lineages. These results suggest that the identified key genes for proinflammatory macrophage, along with the model constructed based on them, possess substantial diagnostic value for T1D patients.

To functionally validate the nine iPMRGs at single-cell resolution, we computed an iPMRG score for each macrophage. The score showed clear subgroup specificity, with mac_C1QB exhibiting the lowest values and the other four T1D-enriched subsets (mac_CCL, mac_APOE, mac_S100A, and mac_MARCO) displaying significantly higher scores (*P* < 0.0001; **Figure 4F**); consistent patterns were observed at the gene level in the DotPlot and on UMAP (**Supplementary Figure 3**; **Figure 4G**). We then defined these four subsets as iPMRGhiMac and mac_C1QB as iPMRGloMac and examined their spatial relationship with other cells using spatial transcriptomics. Notably, in control tissues, iPMRGhiMac signals were preferentially co-localized with endothelial-rich regions, whereas β cells formed well-demarcated, high-abundance clustered islet structures (**Figure 4H**). Whereas, in T1D, iPMRGhiMac signals redistributed away from vascular boundaries and toward the parenchyma, where they became spatial overlap with fragmented, low-abundance residual β-cell signals (**Figure 4H**). CellChat analysis further inferred divergent communication programs: SPP1–ITGAV/ITGB1 signaling was selectively enhanced in the iPMRGhiMac → β cell direction, whereas SST–SSTR2 and MDK–LRP1 were preferentially observed in iPMRGloMac–β cell interactions. Conversely, in the β cell → macrophage direction, DLK1/CNTN1–NOTCH2 signaling was selectively detected toward iPMRGhiMac, and GRN–SORT1 signaling showed higher strength in iPMRGhiMac than in iPMRGloMac. Along the endothelial axis, iPMRGhiMac preferentially engaged endothelial-activating signals such as SPP1 and PRSS1/2–F2R, while endothelial cells reciprocally showed inferred recruitment- and polarization-related signaling toward iPMRGhiMac via ICAM1–ITGAX/ITGB2 and DLL4–NOTCH2 pathways (all *P* < 0.05; **Figure 4I**). Together, these multi-modal analyses support a spatially consistent association between iPMRG-high macrophages and altered local interaction programs involving β cells and endothelial cells in T1D.

### 2.5 Integrated cohorts support the diagnostic value of iPMRGs in peripheral blood

**Figure 5:**
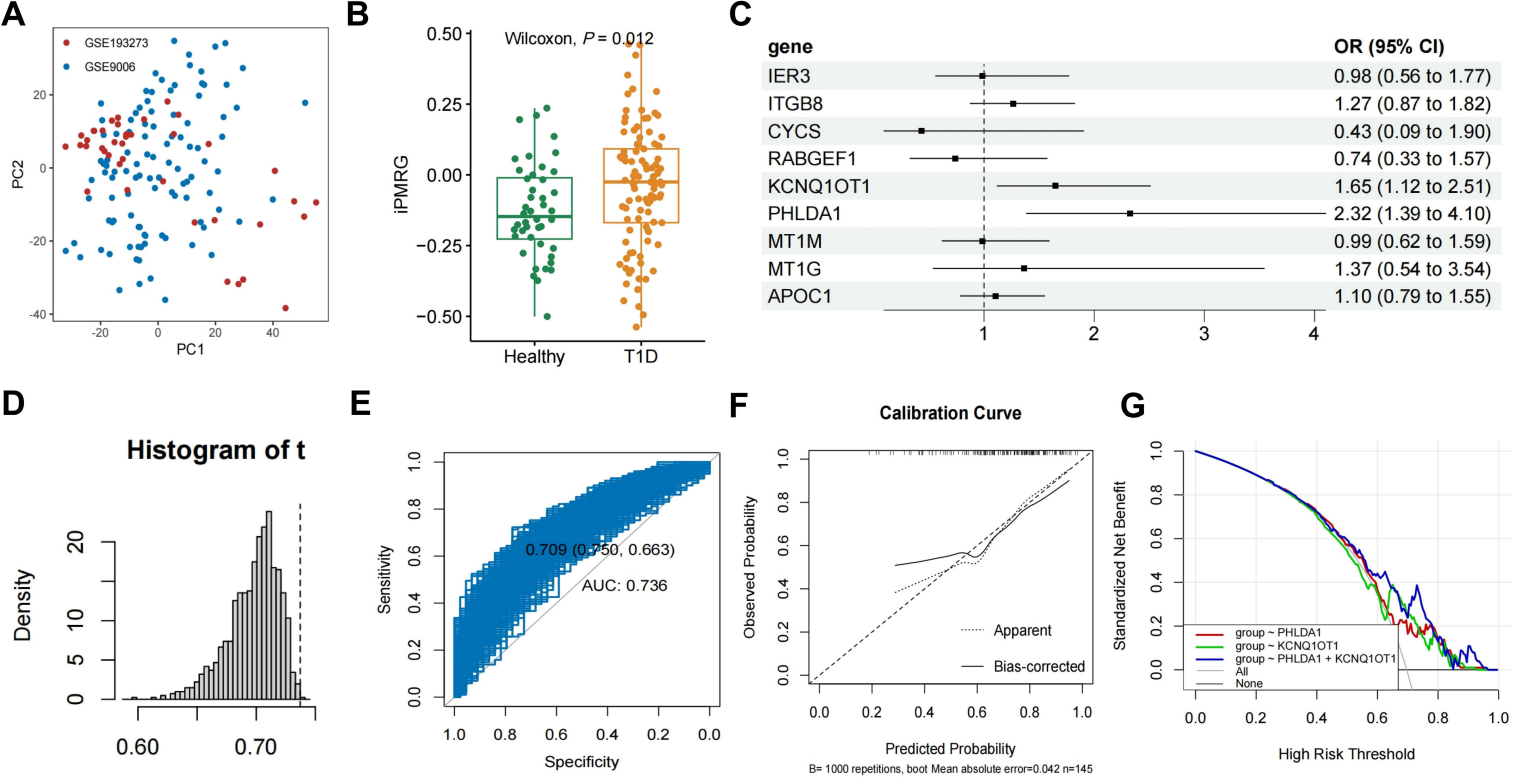
Diagnostic evaluation of iPMRGs in peripheral blood PBMC datasets. **(A)** Integrating two public PBMC datasets (GSE9006 and GSE193273) for GSVA scoring and diagnostic modeling. **(B)** GSVA-based iPMRG scores in T1D versus healthy controls in the combined cohort. **(C)** Multivariable logistic regression results for nine iPMRGs shown as a forest plot. **(D)** Histogram showing the bootstrap distribution of AUC values (1,000 resamples) for the diagnostic model based on iPMRGs. **(E)** Bootstrap-resampled ROC curves (1,000 iterations) for the diagnostic model based on iPMRGs. **(F)** Calibration curve of the diagnostic classifier in the hold-out test set. **(G)** Decision curve analysis evaluating net benefit across threshold probabilities.

To evaluate whether the islet-derived PMRG module is detectable in peripheral blood and supports diagnostic classification, we integrated two public peripheral blood mononuclear cell (PBMC) transcriptome datasets (GSE9006 (35) and GSE193273 (36)) comprising 101 T1D and 44 HC samples (**Figure 5A**). We first quantified iPMRG score using GSVA and observed significantly higher iPMRG scores in T1D compared with HC in the pooled cohort (*P* = 0.012; **Figure 5B**), indicating that the iPMRG-associated inflammatory program is measurable beyond the islet niche.

We next developed a diagnostic classifier based on nine iPMRGs in the integrated PBMC cohort. Univariate logistic regression results for the nine iPMRGs are provided in **Supplementary Table 6**, and multivariable analysis identified PHLDA1 and KCNQ1OT1 as independent diagnostic contributors (**Figure 5C**). The model achieved an apparent AUC of 0.736 on the full cohort. To further assess model robustness, we performed bootstrap validation with 1,000 resamples. This analysis yielded an optimism-corrected AUC of 0.736, with sensitivity of 0.663 and specificity of 0.750 (**Figures 5D, E**). Calibration analysis showed good agreement between predicted and observed probabilities (**Figure 5F**), with a mean absolute error of 0.042 and a Brier score (MSE) of 0.003, reflecting high reliability T1D diagnosis. Decision curve analysis (DCA) demonstrated that the integrated model (blue line) achieved a superior standardized net benefit across a wide range of threshold probabilities, which suggests better potential clinical utility for T1D risk stratification. Collectively, these results support the diagnostic value of iPMRGs in peripheral blood and provide an internally validated PBMC-based classifier for distinguishing T1D from healthy controls.

### 2.6 Peripheral blood PMRG risk score combined with clinical-HLA features predicts IA-to-T1D progression

To further investigate the utility of PMRGs for early risk stratification of T1D, we developed a peripheral blood prediction framework using whole blood RNA-seq data from individuals with islet autoimmunity (IA) in the longitudinal DAISY cohort (37). At the first visit after seroconversion (V1), 137 participants were included for model development and repeated cross-validation. Among 213 whole blood-expressed PMRGs, log-rank analysis identified 18 progression-associated genes, and univariate Cox regression identified 15 genes (**Supplementary Table 7**). Six genes overlapped between the two methods, yielding 27 candidate prognostic PMRGs after taking the union. Exploratory LASSO Cox regression selected 15 core prognostic PMRGs (**Supplementary Figures 4A, B**). This fixed 15-PMRG panel was then used to derive an individual-level PMRG risk score for downstream model evaluation.

**Figure 6:**
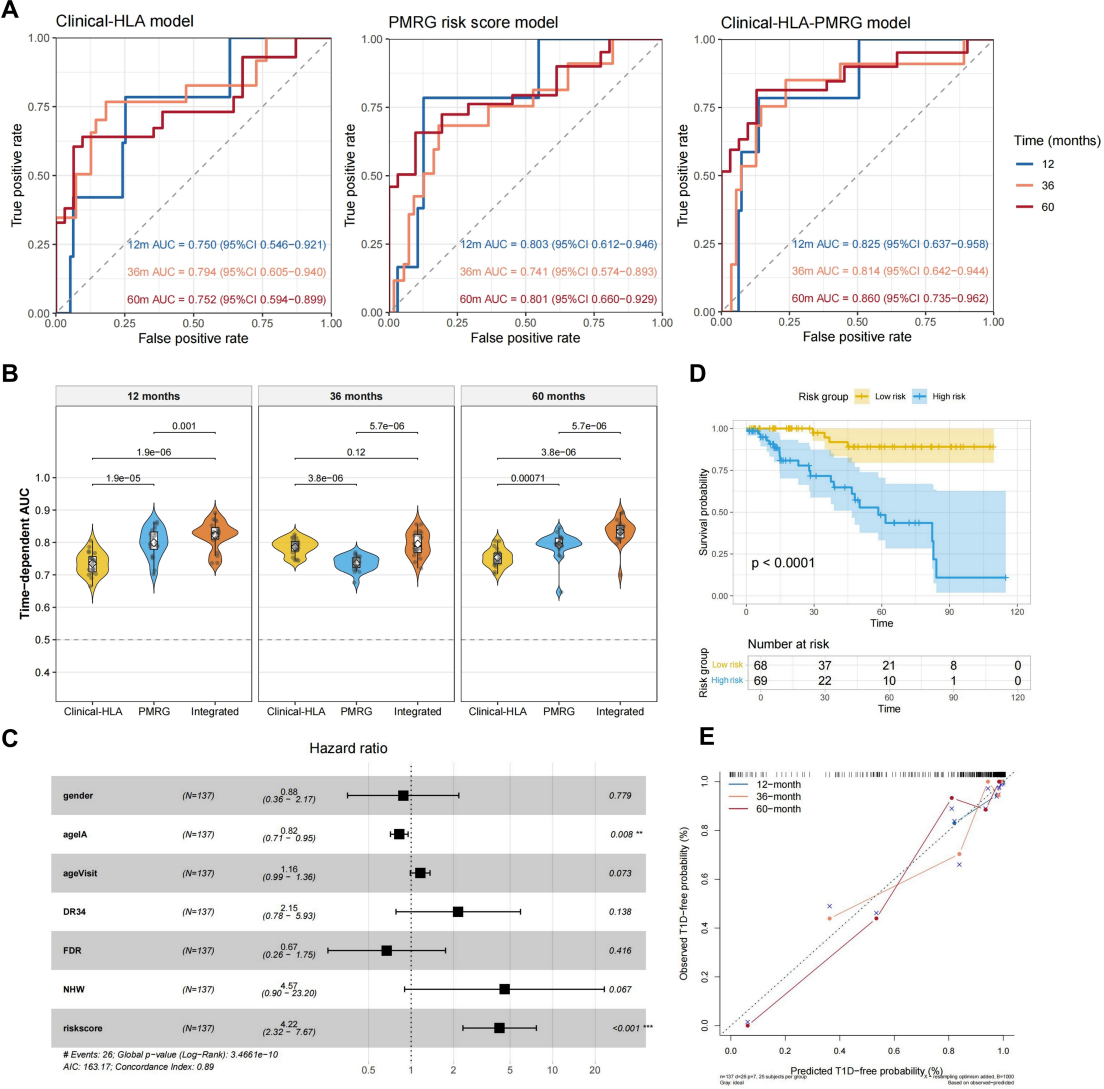
PMRG risk score combined with clinical-HLA features predicts IA-to-T1D progression. **(A)** Time-dependent ROC curves at 12, 36, and 60 months for the the Clinical-HLA model, PMRG risk score model, and the integrated Clinical-HLA-PMRG model. AUC and 95% confidence intervals are shown in the legend. **(B)** Comparison of repeated-CV time-dependent AUCs among the three prediction models. Clinical-HLA, PMRG, and Integrated denote the Clinical-HLA model, PMRG risk score model, and Clinical-HLA-PMRG model, respectively. **(C)** Multivariable Cox proportional hazards analysis for IA-to-T1D progression in the Clinical-HLA-PMRG model, showing hazard ratios (HRs) and 95% confidence intervals for the PMRG risk score and clinical-HLA covariates. **(D)** Kaplan–Meier curves for diabetes-free survival stratified by the final PMRG risk score using the predefined V1-derived cutoff for visualization; log-rank P value and numbers at risk are shown.. **(E)** Calibration curves at 12, 36, and 60 months assessing agreement between predicted and observed risks for the final Clinical-HLA-PMRG display model. ** indicates P < 0.01, *** indicates P < 0.001.

We next compared three predefined prognostic models: the Clinical-HLA model, the PMRG risk score model, and the integrated Clinical-HLA-PMRG model. The Clinical-HLA model achieved AUCs of 0.750, 0.794, and 0.752 at 12, 36, and 60 months, respectively. The PMRG risk score model achieved AUCs of 0.803, 0.741, and 0.801, while the Clinical-HLA-PMRG model achieved AUCs of 0.825, 0.814, and 0.860, respectively (**Figure 6A**; **Supplementary Table 8**). The Clinical-HLA-PMRG model showed consistently higher AUCs than the other two models at most evaluated time points (all *P* < 0.05, except for the integrated model versus Clinical-HLA model in 36 month) (**Figure 6B**). These findings suggest incremental value of the PMRG risk score.

In multivariable analysis, the PMRG risk score remained significantly associated with progression risk after covariate adjustment (HR = 4.22, 95% CI 2.32–7.67, *P* < 0.001; **Figure 6C**), supporting its independent contribution to IA-to-T1D risk stratification. Risk stratification based on the PMRG risk score clearly separated the cumulative incidence curves, with the high-risk group showing a substantially higher risk of progression (log-rank *P* < 0.0001; **Figure 6D**). Calibration analyses at 12, 36, and 60 months demonstrated reasonable agreement between predicted and observed risks, which supports the reliability of the model (**Figure 6E**). Decision curve analysis further indicated that the integrated model provided greater net benefit than “treat-all” and “treat-none” strategies, especially at longer prediction horizons (**Supplementary Figure 4C**). Together, these results support the PMRG risk score as a complementary molecular layer for IA-to-T1D risk stratification when integrated with clinical and HLA information.

### 2.7 Molecular consistency supports generalizability of the prognostic PMRG signature

**Figure 7:**
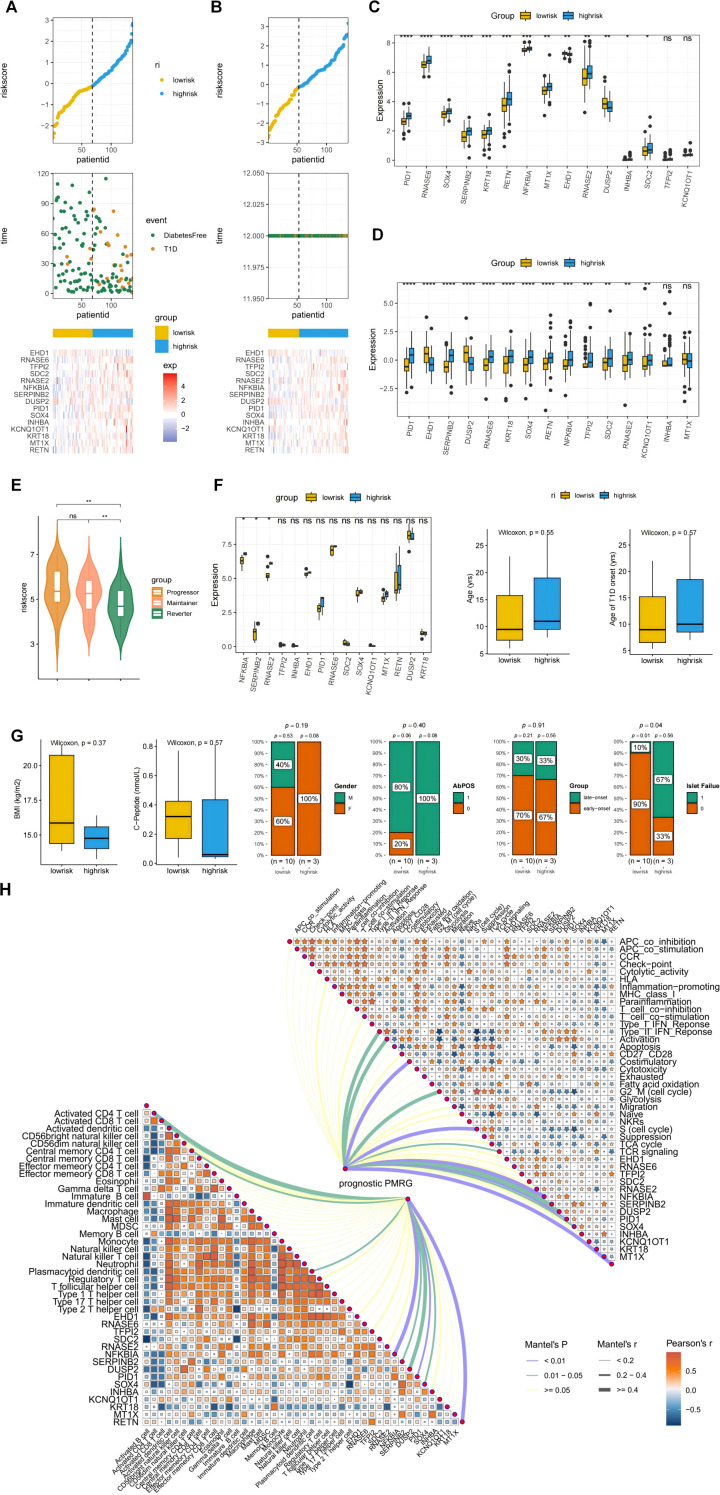
Molecular reproducibility of the prognostic PMRG risk framework. **(A)** Heatmap showing the distribution of PMRG risk scores, IA-to-T1D outcome status, and expression levels of prognostic PMRGs in the V1 cohort. **(B)** Heatmap showing projected PMRG risk scores and prognostic PMRG expression patterns in the V2 cohort. **(C)** Expression differences of prognostic PMRGs between high- and low-risk groups in the V1 cohort. **(D)** Expression differences of prognostic PMRGs between the high- and low-risk groups in the V2 cohort. **(E)** Distribution of projected PMRG risk scores among progressors, maintainers, and reverters in the V2 cohort. **(F)** Expression differences of prognostic PMRGs between projected high- and low-risk groups in the in-house PBMC cohort of patients with type 1 diabetes (T1D). **(G)** Differences in age, age of T1D onset, body mass index (BMI), random C-peptide levels, gender, age of onset classification, islet autoantibody classification, and islet function failure classification between projected risk groups in the in-house PBMC cohort. **(H)** Mantel test-based associations between the prognostic PMRG expression pattern and ssGSEA-derived immune cell compositions, immune pathways and functional scores. * indicates P < 0.05, ** indicates P < 0.01, *** indicates P < 0.001, **** indicates P < 0.0001.

We next examined whether the PMRG risk pattern could be reproduced across longitudinal and independent peripheral blood datasets. In the V1 cohort, ordering samples by the PMRG risk score revealed coordinated expression shifts across the 15 prognostic PMRGs, together with clear separation of high- and low-risk groups and outcome status (**Figure 7A**).

We then projected the final frozen PMRG risk score model to V2 whole blood samples from the DAISY cohort. V2 included 26 progressors who developed T1D during follow-up, 66 maintainers who remained IA, and 45 reverters who returned to a non-autoimmune state. Similar score-associated transcriptomic gradients across prognostic PMRGs were reproduced in V2 (**Figure 7B**). Boxplot comparisons further confirmed consistent gene-level differences in V1 (**Figure 7C**) and V2 (**Figure 7D**), with PID1, RNASE6, SOX4, SERPINB2, KRT18, RETN, NFKBIA, RNASE2, and SDC2 preferentially elevated in the high-risk group, whereas DUSP2 and EHD1 were relatively lower (all *P* < 0.05). Notably, in V2—occurring closer to disease onset—the PMRG risk score differed significantly across outcome-defined groups, with both progressors and maintainers showing higher scores than reverters (*P* < 0.05; **Figure 7E**). These findings support longitudinal reproducibility of the PMRG risk-score pattern.

We further evaluated the prognostic PMRG signature in an independent in-house PBMC cohort of 13 newly diagnosed T1D patients profiled by RNA sequencing. Using the projected PMRG risk score, patients were stratified into high-risk (n = 10) and low-risk (n = 3) groups. The prognostic PMRG expression landscape in this cohort recapitulated the patterns observed in the DAISY datasets (V1/V2), with coordinated shifts across core prognostic PMRGs and significantly higher expression of NFKBIA, SERPINB2 and RNASE2 in the high-risk group (*P* < 0.05; **Figure 7F**). Clinically, the high-risk group showed a higher proportion of islet function failure, whereas BMI, C-peptide, sex, and autoantibody counts were not significantly different between groups (**Figure 7G**).

In addition, Mantel test–based association analyses linked the overall prognostic PMRG expression pattern to ssGSEA-inferred immune cell composition as well as immune functions and pathways. Specifically, the prognostic PMRG gene set showed structured correlations with activated CD4^+^T cells and CD8^+^T cells, together with immune functional signatures including activation, costimulatory, cell cycle and type II IFN response (**Figure 7H**). Collectively, these results support that the prognostic PMRG risk framework captures a reproducible transcriptional signature that aligns with clinically and immunologically relevant phenotypes.

### 2.8 SHAP interpretation reveals model-level contributions and gene-level key PMRG drivers

**Figure 8:**
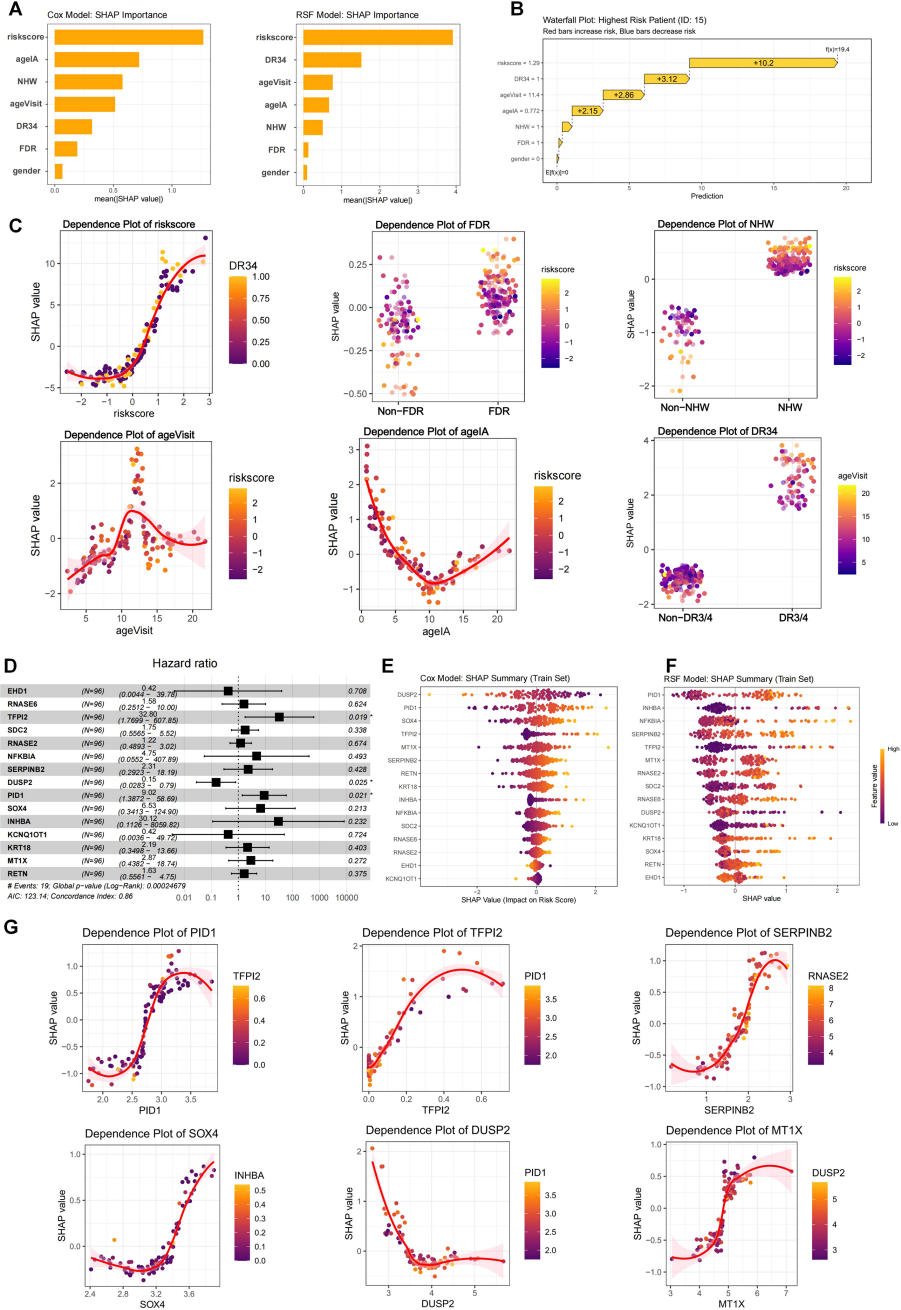
SHAP interpretation reveals model-level risk contributions and key prognostic PMRG drivers. **(A)** SHAP summary plot showing the contribution ranking of the PMRG risk score and clinical-HLA variables in the Clinical-HLA-PMRG Cox and RSF model. **(B)** SHAP waterfall plot showing feature contributions for the highest-risk individual. **(C)** SHAP dependence plots showing the relationships between feature values and SHAP values for the PMRG risk score, age of IA, age at visit, FDR, NHW, and DR3/4. **(D)** Forest plot of the gene-level Cox proportional hazards regression model constructed using the 15 prognostic PMRGs. **(E)** SHAP summary plot showing the contribution ranking of individual prognostic PMRGs in the gene-level Cox model. **(F)** SHAP summary plot showing the contribution ranking of individual prognostic PMRGs in the RSF model. **(G)** SHAP dependence plots showing expression-risk relationships of six key prognostic PMRG drivers and their interaction overlays. * indicates P < 0.05.

To enhance interpretability, we applied SHAP to quantify feature contributions at both the model and gene levels. At the global level, SHAP importance ranking consistently placed the PMRG risk score as the dominant contributors to predicted progression risk in the integrated Clinical-HLA-PMRG model, exceeding the contributions of individual clinical and genetic variables (**Figure 8A**). At the individual level, a SHAP waterfall plot for the highest-risk participant illustrated the cumulative contributions of predictors and highlighted that the PMRG risk score contributed more to the final prediction than genetic background, age, ancestry and family history (**Figure 8B**).

We next interrogated SHAP dependence plots to characterize context-dependent effects. Across the cohort, the PMRG risk score showed a non-linear contribution profile: SHAP values increased sharply beyond a higher-score range, indicating a threshold-like escalation of risk contribution (**Figure 8C**, upper left panel). Notably, interaction analyses suggested that the risk score effect was not uniform across backgrounds. In participants with first-degree family history (FDR) and in non-Hispanic White (NHW) individuals, higher risk score values were accompanied by markedly higher SHAP contributions, consistent with synergistic elevation of predicted risk; in contrast, in non-FDR or non-NHW strata, the same risk score range showed attenuated SHAP contributions, indicating a background-dependent modulation of the molecular risk signature (**Figure 8C**, upper middle and right panels). In contrast, age- and HLA-related features exhibited distinct interaction patterns. Age at visit and age of IA had the strongest contribution within a specific age range, while DR3/4 primarily interacted with age at visit, suggesting that age context shapes how these covariates influence predicted risk (**Figure 8C**, lower panels).

In addition to the composite PMRG risk score, we performed gene-level modeling and interpretability analyses to identify robust molecular drivers of IA-to-T1D progression. In a multivariable Cox regression model including 15 prognostic PMRGs, three genes showed independent prognostic effects. TFPI2 and PID1 were associated with increased progression risk (TFPI2: HR = 32.80, 95% CI 1.77–607.85, *P* = 0.019; PID1: HR = 9.02, 95% CI 1.39–58.69, *P* = 0.021), whereas DUSP2 showed a protective association (HR = 0.15, 95% CI 0.03-0.79, *P* = 0.025) (**Figure 8D**). To prioritize features that are stable across models and datasets, we conducted SHAP analyses for both the Cox and RSF models in the V1 training and V1 validation sets. Genes were ranked by mean absolute SHAP values in each setting, and the intersection of the top contributors consistently identified six key prognostic PMRG drivers (PID1, TFPI2, SERPINB2, SOX4, DUSP2, and MT1X) with stable importance (**Figures 8E, F**; **Supplementary Figures 5A, B**). Notably, the three independent Cox predictors (TFPI2, PID1 and DUSP2) were all contained within this six-gene consensus set, supporting convergence between inferential (Cox) and predictive (SHAP) evidence. SHAP dependence plots for six key prognostic PMRG drivers further revealed non-linear expression–risk relationships while showing limited interaction effects in the overlays (**Figure 8G**), indicating that their contributions are largely stable across the observed expression ranges. Together, these multi-level interpretability results clarify how the model integrates transcriptome-derived risk with clinical and genetic context, and they provide a practical basis for deploying a peripheral blood–based risk assessment tool to stratify IA individuals for intensified monitoring and early intervention.

### 2.9 Experimental validation of key PMRGs

**Figure 9:**
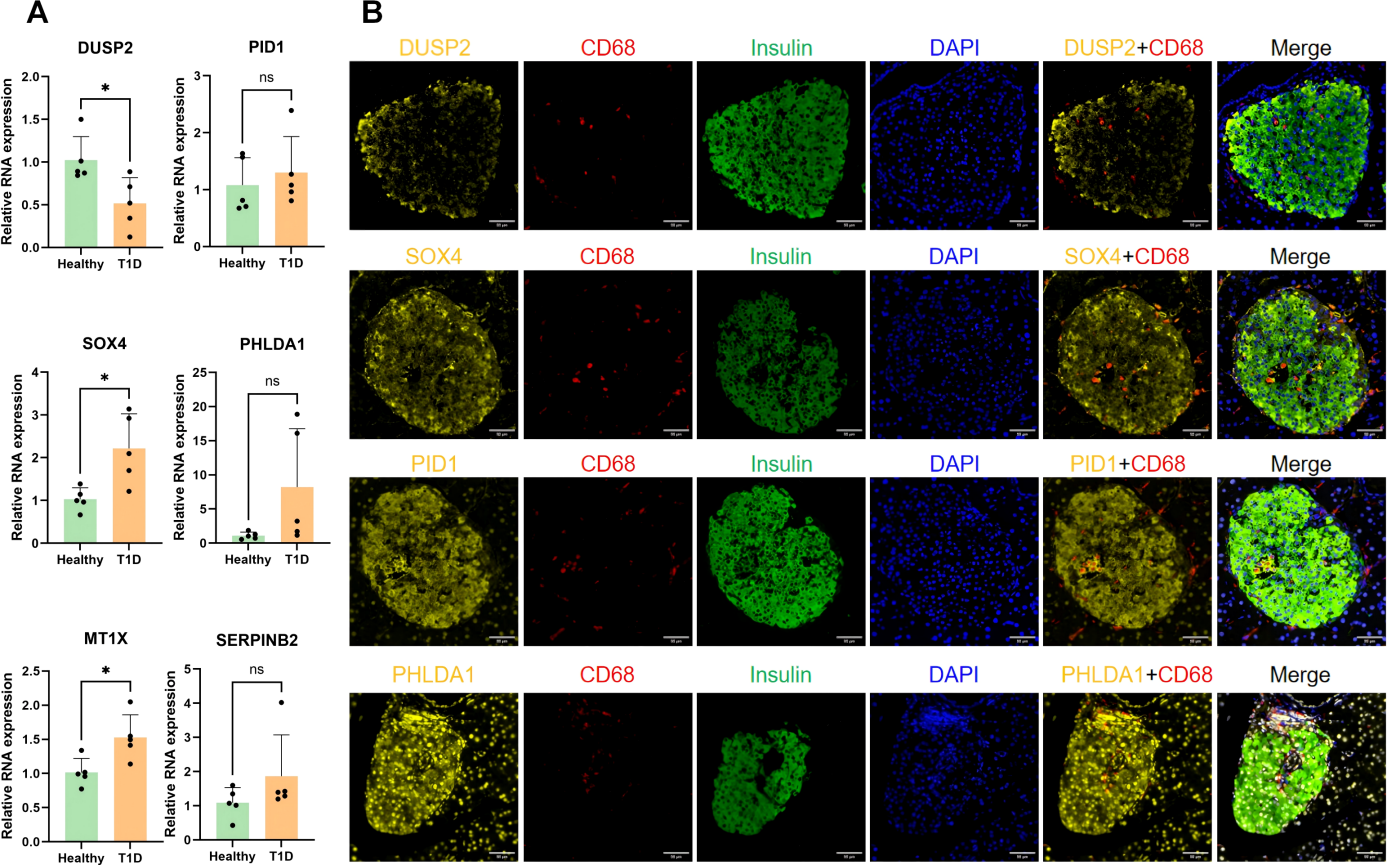
Experimental validation of key PMRG-related genes in independent PBMC samples and mouse pancreatic tissues. **(A)** RT-qPCR analysis of DUSP2, SOX4, MT1X, PID1, SERPINB2, and PHLDA1 in PBMCs from 5 T1D patients and 5 healthy controls. **(B)** Multiplex immunofluorescence staining showing the distribution of selected PMRGs together with Insulin and CD68 in mouse pancreatic tissues. SOX4, PID1, and PHLDA1 staining was performed in NOD mouse pancreatic sections, whereas DUSP2 staining was performed in ICR mouse pancreatic sections. Yellow indicates DUSP2, SOX4, PID1, or PHLDA1; red indicates CD68; green indicates Insulin; blue indicates DAPI. Scale bar, 50 μm. Data are shown as mean ± SD. *P* values were calculated using two-tailed t tests. ns indicates not significant; * indicates P < 0.05.

To provide additional validation for the key genes identified by transcriptomic analysis and machine-learning selection, we first performed RT-qPCR in an independent PBMC cohort comprising 5 T1D patients and 5 healthy controls. As shown in **Figure 9A**, SOX4 and MT1X were significantly upregulated in T1D, whereas DUSP2 was significantly downregulated. PID1, SERPINB2, and PHLDA1 showed concordant directional changes with the transcriptomic results, although the differences did not reach statistical significance. These findings provide partial independent support for the PBMC expression patterns of key PMRGs.

We next performed multiplex immunofluorescence staining in mouse pancreatic tissue sections to examine the tissue distribution of selected markers together with CD68 and Insulin (**Figure 9B**). In NOD mouse islets, SOX4, PID1, and PHLDA1 signals were detectable within the islet region and showed spatial overlap or close adjacency with subsets of CD68^+^ cells. In contrast, clear co-localization of DUSP2 with CD68^+^ cells was not observed in NOD islets, whereas partial spatial overlap was detected in ICR mouse islets. These results support the tissue relevance of the selected markers while suggesting that individual PMRGs may exhibit context-dependent distribution patterns within the islet inflammatory microenvironment.

## 3 Discussion

Despite the availability of islet autoantibodies for risk stratification, accurately predicting progression from islet autoimmunity to clinical T1D remains a major unmet need (38). In the present study, we integrated islet bulk RNA-seq, single-cell and spatial transcriptomic data with peripheral blood transcriptomic profiling and machine learning to delineate macrophage remodeling within the pancreatic islet and to translate tissue-derived inflammatory signatures into accessible peripheral blood biomarkers. Collectively, our findings connect three levels of evidence: (i) islet immune remodeling with myeloid enrichment and inflammatory macrophage heterogeneity, (ii) a pro-inflammatory macrophage trajectory linked to a compact islet-derived PMRG signature, and (iii) peripheral blood-based diagnostic and time-to-event risk models with interpretable drivers. Together, these results provide a macrophage remodeling-centered, tissue-to-blood framework for early risk assessment in T1D.

First, our integrated analysis of bulk and single-cell RNA-seq data revealed marked differences in transcriptomic profiles and immune cell composition between T1D and healthy islets. We observed substantial myeloid enrichment within the T1D islet microenvironment, which is consistent with previous studies implicating innate immune activation in disease progression (39). At single-cell resolution, this broad inflammatory shift resolved into five distinct macrophage states with clear functional divergence. Among them, mac_C1QB was relatively more abundant in control islets and retained features more consistent with a homeostatic macrophage state (40), whereas mac_APOE, mac_S100A, mac_MARCO, and mac_CCL were enriched in T1D and were operationally grouped as proinflammatory macrophages for subsequent analyses. Importantly, these proinflammatory subgroups were not identical: mac_APOE retained prominent metabolic and biosynthetic features, mac_S100A was characterized by complement-associated inflammatory programs, mac_MARCO combined terminal complement and antimicrobial signatures, and mac_CCL showed preferential enrichment of innate inflammatory sensing and effector-associated pathways, and its GSVA profile was more strongly defined by NOD-like receptor signaling, interleukin-1 processing, chemokine receptor binding, and prostaglandin/thromboxane synthesis. Together, these findings indicate that inflammatory macrophage remodeling in T1D islets is composed of multiple related but non-equivalent states rather than a single uniform phenotype. This pattern cannot be fully explained by a simple M1/M2 classification (41), but instead reflects a set of disease-associated states shaped by the inflamed islet microenvironment (17, 42).

Pseudotime trajectory analysis further added a dynamic dimension to these observations by suggesting progression from the more homeostatic mac_C1QB state toward multiple disease-enriched inflammatory states, including mac_CCL, mac_APOE, mac_S100A, and mac_MARCO. This shift was accompanied by coordinated upregulation of pathways related to inflammatory sensing, leukocyte recruitment and activation, antimicrobial responses, and apoptotic signaling, together with downregulation of pathways involved in antigen processing and presentation, lymphocyte differentiation, cytokine regulation and responsiveness, and mononuclear cell migration. Importantly, these trajectory-level changes describe the overall direction of macrophage remodeling toward multiple inflammatory states, rather than the complete static phenotype of any single subgroup. In this setting, late-stage macrophage remodeling in T1D islets is best understood as a proinflammatory but functionally non-classical state, in which inflammatory amplification coexists with attenuation of selected APC- and cytokine responsiveness-related functions. Collectively, these changes suggest stronger inflammatory effector functions alongside weaker surveillance- and homeostasis- related activities (16, 43), which supports a model in which inflammatory amplification and reduced regulatory restraint together characterize macrophage reprogramming in T1D. This interpretation is broadly consistent with recent reviews emphasizing that islet macrophages contribute to both immune homeostasis and diabetes-associated inflammatory remodeling across disease stages (44). Our spatial and communication analyses further placed this trajectory in anatomical context. In control tissue, inflammatory macrophage-associated signals were preferentially aligned with endothelial-rich regions, whereas in T1D they redistributed toward parenchymal areas containing fragmented residual β-cell signals. Together with CellChat inference, these findings suggest altered local microenvironmental communication programs among proinflammatory macrophages, β cells, and endothelial cells. Although these analyses do not establish causality, they provide exploratory support for a niche-level framework in which inflammatory macrophage states, endothelial interfaces, and stressed β-cell remnants are spatially linked in a manner that may facilitate immune-cell recruitment, retention, and inflammatory priming (45). Importantly, this state heterogeneity provides a mechanistic basis for why a concise macrophage-related inflammatory program can be detected in peripheral blood and retain diagnostic and prognostic value in downstream models.

A major translational contribution of this work is the development of peripheral blood–based models for T1D diagnosis and prognosis. In the diagnostic setting, we showed that the islet-derived PMRGs is detectable in PBMC transcriptomes, indicating that disease-relevant inflammatory programs can be captured in accessible blood samples. This should not be interpreted as a direct surrogate of islet cellular composition; rather, PBMC readouts likely reflect a shared systemic proinflammatory program associated with pancreatic inflammation (46). On this basis, we developed an iPMRG-based diagnostic classifier that distinguished T1D from healthy controls with supportive internal validation, calibration, and decision-curve analyses, supporting its potential value as a noninvasive immune-state readout. The appearance of PHLDA1 and KCNQ1OT1 as independent contributors adds biological plausibility to the diagnostic model, although the strength of current evidence differs between them. In particular, the concordant directional change of PHLDA1 in the independent PBMC RT-qPCR validation provides additional support for its relevance in the peripheral inflammatory context. Existing studies indicate that PHLDA1 participates in inflammatory signaling and innate immune regulation across multiple disease contexts, including macrophage- or microglia-associated inflammatory responses, suggesting that its elevation may reflect a disease-associated peripheral immune context (47–49). By contrast, KCNQ1OT1 has been linked to immune regulation in tumor immunology (50), but direct evidence in T1D or macrophage remodeling remains limited, and its contribution should therefore be interpreted with caution. The limited overlap between the diagnostic and prognostic signatures, with only KCNQ1OT1 shared, is nevertheless biologically plausible because the two models were optimized for related but distinct tasks: the diagnostic model was designed to distinguish established T1D from healthy controls, whereas the prognostic model was intended to capture progression-related variation within the IA-to-T1D continuum. Under this framework, most genes would be expected to be phase-specific, while the persistence of KCNQ1OT1 across both signatures may indicate a shared regulatory component that spans overt disease classification and preclinical progression, albeit with limited mechanistic evidence at present.

More importantly, in the IA setting, the 15-gene PMRG risk score provided a time-to-event framework for predicting progression to clinical T1D, and integration of this score with clinical and HLA context improved discrimination over a PMRG risk score-only model. This complements current risk stratification approaches based on autoantibody profiles (4), genetic risk scores (3), and age at seroconversion (5). Islet autoantibody type and number remain among the most informative predictors of preclinical progression (51, 52), whereas T1D-GRS based approaches support early screening and stratification, although their performance varies across populations and requires ancestry-aware calibration (53). However, both marker classes have important limitations. Autoantibody positivity marks the initiation of β-cell loss and therefore may not represent the earliest biomarker of disease progression, whereas GRS is intrinsically static and provides only a single lifetime risk estimate (54). In this context, the PMRG framework may help address part of this gap by capturing macrophage-related proinflammatory activity as a dynamic molecular layer that is not fully reflected by conventional serological or genetic markers alone (37, 55, 56). Because the present dataset did not include complete individualized autoantibody panel data or a directly comparable PRS/T1D-GRS measure, we could not perform a formal head-to-head comparison. Nevertheless, our findings are conceptually aligned with the emerging view that newer dynamic risk scores may enhance static biomarkers for predicting progression and for evaluating disease-modifying interventions (6, 7).

Interpretability analyses provide additional support for the biological and clinical relevance of this framework. SHAP consistently identified the PMRG risk score as the dominant predictor of progression and highlighted a set of stable gene-level contributors across different modeling paradigms. Among these genes, DUSP2 is particularly notable because multiple lines of evidence in this study consistently supported its relevance. In PBMCs, DUSP2 was significantly downregulated in T1D, and in tissue-level staining it showed overlap with subsets of CD68+ cells in ICR islets, whereas clear co-localization was not observed in NOD islets. Together with prior evidence that DUSP2 participates in immune-cell signaling and autoimmune regulation (57), these findings support the possibility that loss of DUSP2-related immune restraint may accompany progression toward a more proinflammatory state. SOX4 and MT1X were significantly increased in independent PBMC samples, suggesting that the prognostic PMRG framework captures not only inflammatory activation but also stress-adaptive remodeling programs. This interpretation is broadly consistent with recent evidence linking SOX4 to immune-related transcriptional regulation (58, 59), and with recent reviews emphasizing that metallothioneins, including MT1X, are involved in oxidative stress responses and immune regulation (60). In addition, PID1 and SOX4 were detectable in inflamed islet regions adjacent to CD68+ cells, supporting its tissue-level relevance. Although SERPINB2 did not reach statistical significance in PBMC RT-qPCR, its concordant directional trend remains compatible with its previously noted role in macrophage-associated inflammatory regulation (61–63). Together, these observations suggest that progression-related risk in our model is biologically grounded in a composite immune program. The background-dependent contribution patterns observed in SHAP dependence analyses further suggest that the effect of the molecular risk program may vary across subgroups, such as those defined by family history or ancestry, with direct implications for model portability and personalized calibration. Together with the comparable performance of the RSF model, these findings support the robustness of the overall predictive framework and its potential utility for context-aware risk stratification in heterogeneous populations.

This study has several strengths. First, we applied a tissue-to-blood design that anchors peripheral biomarkers in mechanistically relevant inflammatory programs derived from the pancreatic lesion. Second, we integrated complementary data modalities—bulk islet transcriptomics, single-cell profiling, spatial context, and experimental validation—to strengthen the biological plausibility of the identified PMRG framework and reduce ambiguity in cell-type attribution. Third, the predictive framework was assessed using discrimination, calibration, and decision-curve analysis, and its robustness was further supported by concordant performance across linear and non-linear modeling paradigms. Finally, the explicit use of SHAP improved interpretability and yielded a compact set of stable gene-level contributors that may facilitate both downstream mechanistic studies and future assay simplification.

Several limitations should also be acknowledged. First, sample sizes were modest in key parts of the study, particularly the in-house cohort, which may have reduced statistical power and limited generalizability. Second, although independent PBMC RT-qPCR and tissue-level immunofluorescence provided additional experimental support, most evidence in this study remains computational and associative. The validation was limited in scale and scope: the independent PBMC cohort was small, only a subset of tested genes reached statistical significance, and the tissue-level validation was performed in mouse rather than human pancreatic tissue. In addition, not all markers showed the same spatial distribution pattern, suggesting context-dependent expression. Likewise, spatial co-localization and CellChat-inferred communication patterns should therefore be interpreted as supportive rather than causal evidence, and further functional studies are still required to establish causal roles for islet-derived and prognostic PMRG genes in macrophage remodeling and β-cell injury. Third, although we performed repeated-CV internal validation, V2 longitudinal projection, and exploratory assessment in an in-house PBMC cohort, the validation hierarchy should be interpreted cautiously. The V2 analysis represents within-cohort reproducibility rather than fully external validation, and the in-house PBMC cohort served as an exploratory molecular consistency check because it lacked event-time annotations and matched controls. Therefore, prospective validation in larger, multi-center, and ethnically diverse cohorts remains essential. Fourth, insulin therapy and chronic hyperglycemia may also have influenced transcriptional patterns in the established T1D case-control datasets, although this concern is less relevant to the DAISY prognostic cohort because the V1 and V2 samples were obtained during stages 1–2 islet autoimmunity before clinical diabetes. Fifth, integrating transcriptomic data from different platforms and sources may have introduced residual batch effects, which could affect coefficient portability and threshold transferability. Future studies should therefore use more standardized assay workflows, such as targeted expression panels, to improve clinical applicability. Finally, the upstream factors that trigger inflammatory macrophage remodeling and the downstream pathways linking these macrophage states to β-cell loss are still not fully understood and require further mechanistic study.

In conclusion, our study improves the understanding of macrophage heterogeneity and remodeling in T1D and supports a central role for inflammatory macrophage programs in disease pathogenesis. By identifying biologically grounded islet-derived and prognostic PMRG signatures, we translate tissue-based immune insights into peripheral blood diagnostic and prognostic tools. These findings not only provide insight into macrophage-driven immunopathology in T1D, but also establish a practical framework for early risk stratification, precision monitoring, and future prevention strategies in autoantibody-positive individuals.

## 4 Materials and methods

### 4.1 Subjects

We recruited 18 newly diagnosed T1D patients and 5 healthy controls at The First Affiliated Hospital with Nanjing Medical University in July 2023. All T1D individuals completed a structured questionnaire and underwent a physical examination, during which we collected data on age, gender, age of T1D onset, treatment modalities, height, and weight. Each participant provided a 5 mL blood sample. PBMCs were isolated from the samples via density gradient centrifugation using Lymphoprep lymphocyte separation medium (Fresenius KabiNorge AS, Norway). Participants with T1D were also assessed for islet autoantibodies and random serum C-peptide levels. Islet autoantibodies were measured using a Yahuilong 3000 iFlash chemiluminescence immunoassay (Shenzhen Yahuilong Biotechnology, China). Serum C-peptide levels were measured by chemiluminescence (Roche Diagnostics, Switzerland). Early-onset T1D was defined as diagnosis before the age of 14, while late-onset T1D was defined as diagnosis thereafter. The antibody-positive group was characterized by the presence of at least one islet autoantibody, while the islet function failure group was defined by a random serum C-peptide level below 0.1 nmol/L. Baseline characteristics of the 13-case in-house PBMC RNA-seq cohort and the independent PBMC validation cohort comprising 5 T1D patients and 5 healthy controls are summarized in **Supplementary Tables 9**, **10**, respectively. Written informed consent was obtained from all participants, and the Ethics Committee of the First Affiliated Hospital with Nanjing Medical University approved this study.

### 4.2 RNA sequencing and data processing

Total RNA was extracted from PBMCs, and library preparation was conducted according to the Illumina standard protocol (VAHTS Universal V6 RNA-seq Library Prep Kit for Illumina). Agilent 4200 Bioanalyzer was utilized to assess the concentration and size distribution of cDNA library prior to sequencing on the Illumina NovaSeq 6000. The high-throughput sequencing followed the manufacturer’s instructions precisely. The raw reads were filtered by Seqtk before mapping to genome using Hisat2 (version 2.0.4) (64). Gene fragments were quantified using StringTie (version 1.3.3b) and normalized with the trimmed mean of M values (TMM) (65).

### 4.3 Data acquisition

Bulk RNA sequencing (RNA-seq) datasets of pancreatic islet samples were obtained from the GEO database [GSE181674 (22), GSE50244 (23), and GSE162689 (24)], including 14 samples from type 1 diabetes (T1D) patients and 67 samples from healthy controls. For single-cell RNA sequencing (scRNA-seq) analysis, human islet scRNA-seq data were retrieved from the Human Pancreas Analysis Program Database (66), comprising 8 T1D patients and 8 healthy controls. Spatial transcriptomics data for pancreatic tissue were obtained from GSE296626 (67), including one T1D sample and one healthy control sample.

Bulk transcriptome datasets of peripheral blood mononuclear cells (PBMCs) for T1D versus healthy controls were retrieved from public repositories [GSE9006 (35) and GSE193273 (36)]. For IA-to-T1D time-to-event modeling, we used the DAISY longitudinal islet autoimmunity (IA) cohort (GSE230370 (37), n = 274). Whole blood samples were collected at V1 (the first visit after seroconversion) and V2 (the last visit before disease onset). Progression to clinical T1D was defined by the original study criteria (37). For analyses without event-time annotations, V2 samples were categorized as progressors (subsequently developed T1D), maintainers (remained IA-positive), or reverters (returned to a non-autoimmune state). All datasets were publicly available, and ethical approvals and informed consents were obtained by the original studies.

A detailed summary of all public datasets, including accession number, biological source, retained samples, disease groups, and analytical purpose, is provided in **Supplementary Table 11**.

### 4.4 Bulk and single-cell RNA-seq data processing

The bulk RNA-seq data from islet samples were processed using R software (version 4.2.0). Quality control and normalization were conducted before downstream integration. Shared genes across datasets were retained and the expression matrices were merged. Batch correction for source effects was then performed using the ComBat function in the “sva” package. For integrated PBMC transcriptomic datasets used for diagnostic modeling, shared genes were retained, expression matrices were merged, and batch corrected was performed using a similar strategy. PCA plots before and after batch correction are shown in **Supplementary Figures 6A–D**. Differential expression analysis was subsequently performed using the “limma” package (version 3.54.2). Genes with an adjusted p-value < 0.05 and an absolute log2 fold change (|log2FC|) > 1 were classified as differentially expressed genes (DEGs). Batch correction was applied only to merged datasets used for integrated analyses. Independent cohorts used for prognostic modeling or exploratory validation were analyzed separately without batch correction.

ScRNA-seq data were processed using the “Seurat” package (version 4.3.0.1) in R software. Cells were filtered out if they expressed fewer than 1,000 genes or if the mitochondrial gene content exceeded 15%. After normalization, 3,000 highly variable genes were selected, followed by scaling and principal component analysis (PCA). To reduce donor-level batch effects, batch correction was performed using the “Harmony” package with hpap_id, and the first 30 corrected principal components were used for downstream clustering and visualization. Uniform Manifold Approximation and Projection (UMAP) was employed using the Harmony reduction, and cell clustering was conducted using the FindNeighbors and FindClusters (resolution = 1.5) functions. UMAP plots after batch correction are shown in **Supplementary Figure 1A**.

### 4.5 Cell type annotation

Cell clusters were annotated based on canonical marker genes from the CellMarker database (68), and additional marker genes identified for each cluster using the FindAllMarkers function. A total of 12 major cell types were identified, including α cells, β cells, δ cells, γ cells, proliferating endocrine cells, acinar cells, quiescent and activated stellate cells, endothelial cells, ductal cells, Schwann cells, and immune cells.

Immune cells expressing PTPRC (CD45) were selected for further dimensionality reduction and clustering analysis. Utilizing cell type-specific marker genes reported in the literature (29–31), subsequent annotation revealed six major immune cell types, including macrophage subtypes (mac_APOE, mac_S100A, mac_MARCO, mac_CCL, and mac_C1QB), and mast cells. Subgroup-specific marker genes were identified using the FindAllMarkers function in Seurat. GO and KEGG enrichment analyses of macrophage subgroup-specific genes were performed using the clusterProfiler package. To further compare pathway-level programs across macrophage subgroups, curated GSVA was performed using KEGG and REACTOME gene sets. Based on M1 and M2 characteristic gene sets reported in the literature (32), we employed the AddModuleScore function in the Seurat package to calculate the M1 score and M2 score for macrophage subgroups. In addition, an iPMRG score was computed based on the nine iPMRG genes to quantify the islet-derived proinflammatory macrophage signature across macrophage subgroups.

### 4.6 Donor-level robustness analysis

To reduce the risk of pseudoreplication in single-cell analyses, macrophage composition was recalculated at the donor level (**Supplementary Table 12**). Donor-level proportions of macrophage subgroups and grouped proinflammatory/homeostatic macrophage states were compared between control and T1D samples. Leave-one-donor-out sensitivity analyses were performed by excluding one donor at a time and repeating the compositional comparisons.

### 4.7 Pseudotime trajectory analysis

Pseudotime trajectory analysis was conducted using the “monocle” package (version 2.18.0) (33) to infer the differentiation pathways of macrophages. Cells were ordered along pseudotime to model the progression of macrophage remodeling. DEGs along the trajectory were identified and clustered into two modules based on similar expression dynamics. A total of 265 genes in the upregulated module were refined as proinflammatory macrophage-related genes (PMRGs) for subsequence analyses.

### 4.8 Machine learning for diagnostic feature selection

For diagnostic feature selection in islet bulk transcriptomes, the 265 PMRGs were used as candidate predictors. GSE181674 (22) and GSE50244 (23) were combined as the training set, while GSE162689 (24) served as the external validation set. Univariate logistic regression was first performed to screen diagnostic candidates, followed by the Least Absolute Shrinkage and Selection Operator (LASSO) logistic regression (glmnet, version 4.1-8) with ten-fold cross-validation to select the optimal penalty parameter (λ). The resulting nine islet-derived PMRGs (iPMRGs) were defined as the final diagnostic gene signature for T1D.

Subsequently, seven different machine learning algorithms (logistic regression, iterative dichotomizer 3, random forest, support vector machines, naive Bayes classifier, recursive partitioning and regression trees, k nearest neighbors) were applied using the “mlr3” package (version 0.20.2), and model hyperparameters were optimized by five repeated ten-fold cross-validation. Model performance was evaluated by area under the curve (AUC) and related classification metrics (sensitivity, specificity, false negative, and false positive rates), and the Naive Bayes model was selected as the final classifier based on the best overall performance in the validation set.

### 4.9 Functional annotation and immune cell infiltration analysis

For bulk RNA-seq, DEGs were functionally annotated using Gene Ontology (GO) and Kyoto Encyclopedia of Genes and Genomes (KEGG) enrichment analyses with the “clusterProfiler” package (version 4.9.3.002) (69). Gene Set Enrichment Analysis (GSEA) (70) was conducted to identify enriched pathways, with thresholds set at an adjusted p-value < 0.05, |Normalized Enrichment Score (NES)| > 1, and a false discovery rate (FDR) q-value < 0.25. The CIBERSORT algorithm (25), utilizing the LM22 signature matrix and 1,000 permutations, was applied to estimate the proportions of immune cell types in samples from islets. Single-sample gene set scoring was performed using the GSVA package (version 1.46.0). Immune cell type, pathway and function scores (27, 28) were estimated using the ssGSEA (26) method, while PMRGs score (265 genes) and iPMRG score (nine genes) was quantified using the GSVA (34) method.

For islet single-cell analyses, five macrophage subtypes were defined as iPMRGhi and iPMRGlo groups based on subgroup identity and iPMRG score distribution. Cell–cell communication among iPMRGhi macrophages, iPMRGlo macrophages, β cells, and endothelial cells was inferred using CellChat (version 2.2.0) with default settings, and pathway-level interaction strengths and representative ligand–receptor pairs were summarized. Exploratory spatial analysis and microenvironmental inference were performed using spatial transcriptomics to map macrophage, endothelial, and endocrine signatures and to examine the spatial distribution of iPMRGhi and iPMRGlo macrophage signals relative to β-cell markers.

### 4.10 Construction of diagnostic models in peripheral blood

For PBMC diagnostic modeling, a diagnostic classifier was constructed using logistic regression with the nine iPMRG genes as predictors. Univariate logistic regression was performed using the glm function in R, and multivariable logistic regression was used to identify independent diagnostic contributors. Model discrimination was assessed by ROC analysis and AUC. Internal validation was performed using 1,000 bootstrap resamples to generate the empirical distribution of AUC and to summarize performance across resamples (e.g., sensitivity and specificity). Calibration curves were generated using the ‘rms’ package (version 6.7-1) to assess agreement between predicted and observed probabilities, and decision curve analysis (DCA) was conducted using the ‘rmda’ package (version 1.6) to evaluate potential clinical utility across threshold probabilities.

### 4.11 Construction of predictive models in peripheral blood

For IA-to-T1D progression prediction, whole blood RNA-seq data were obtained from the longitudinal IA cohort [GSE230370 (37)]. A total of 213 whole blood-expressed PMRGs were first screened using log-rank tests and univariate Cox regression. PMRGs with P < 0.05 in either analysis were combined as candidate prognostic genes and entered into an exploratory LASSO Cox regression using the ‘glmnet’ package (version 4.1-8). This feature-reduction step identified a fixed 15-PMRG panel for subsequent risk-score construction and model evaluation. The PMRG risk score was calculated as a weighted sum of the 15 prognostic PMRGs’ standardized expression values multiplied by their corresponding coefficients.

To evaluate the prognostic contribution of the PMRG risk score, three Cox proportional hazards models were constructed using the ‘survival’ package (version 3.5-7). The Clinical-HLA model included predefined clinical variables (gender, age of IA, age at visit, ancestry, family history) and HLA phenotype. The PMRG risk score model included the PMRG risk score alone. The Clinical-HLA-PMRG model combined the PMRG risk score with the clinical and HLA variables.

Formal model discrimination was assessed using repeated cross-validation with out-of-fold prediction. In each repeat, the V1 dataset was split into training and testing folds while preserving the event distribution. model fitting, scaling, tuning, and coefficient estimation were performed within the training folds, and predictions were generated for the held-out testing folds. Out-of-fold predictions from all repeats were combined to estimate time-dependent AUCs at 12, 36, and 60 months using the ‘timeROC’ package (version 0.4). Bootstrap 95% confidence intervals were calculated for all AUC estimates. Incremental value was evaluated by comparing the Clinical-HLA-PMRG model with the other two models using AUCs. Event numbers and censoring patterns were summarized at each evaluation time (**Supplementary Table 13**).

After repeated cross-validation, final display models were fitted using the full V1 dataset. Risk stratification for Kaplan–Meier analysis was performed using the PMRG risk score, and the log-rank test was used to compare diabetes-free survival between risk groups. Calibration curves at 12, 36, and 60 months were plotted using the ‘rms’ package (version 6.7-1). Clinical utility was assessed using decision curve analysis (DCA) with the ‘ggDCA’ package (version 1.2). The PMRG risk score was further used to visualize transcriptomic risk gradients by ordering samples and displaying heatmaps together with the PMRG risk score, outcome status, and prognostic PMRG gene expression levels.

To assess robustness under a non-linear modeling framework, an random survival forest (RSF) model was constructed using randomForestSRC (version 3.5.0) with the same predictor set. Model interpretation was further supported by SHAP-based analyses to examine the relative contribution of the PMRG risk score, clinical and HLA variables in the integrated model.

For gene-level model interpretation, V1 samples were additionally divided into a stratified 70% training subset and a 30% testing subset. Gene-level Cox and RSF models were fitted using the 15 PMRGs, and SHAP analyses were performed to examine the relative contribution and stability of individual PMRGs across modeling paradigms.

Additional evaluation of the PMRG risk score was performed using V2 whole blood samples from the DAISY cohort. Projected PMRG risk scores in V2 were calculated using the final frozen model derived from V1, applying the same 15-PMRG panel, scaling parameters, and Cox regression coefficients. The V2 dataset was used to examine longitudinal reproducibility of the PMRG risk-score pattern.

An in-house PBMC cohort of 13 newly diagnosed T1D patients was used for exploratory molecular consistency assessment. Projected PMRG risk scores were calculated using the final frozen V1 model. Clinical characteristics (including gender, age of T1D onset, age at visit, BMI, islet autoantibodies, and random serum C-peptide levels) were summarized according to projected PMRG risk patterns. Associations between the PMRG-related expression pattern and ssGSEA-derived immune score matrices were evaluated using Mantel tests.

### 4.12 Model interpretability analysis (SHAP)

Shapley additive explanations (SHAP) were used to interpret both the integrated Cox and RSF prediction models (‘fastshap’ package, version 0.1.1; ‘shapviz’ package, version 0.10.3). Global-level SHAP summary plots were generated to rank the contributions of the PMRG risk score and clinical/genetic covariates, and individual-level attributions were visualized using waterfall plots for the highest-risk participant. SHAP dependence plots were used to assess non-linear contribution patterns and to visualize context-dependent effects by stratifying interaction variables (e.g., FDR, NHW, age-related and HLA-related features). For gene-level interpretation, SHAP values were computed for individual prognostic PMRG genes, and prioritized drivers were defined by intersecting the top six genes ranked by mean absolute SHAP values across model types (Cox and RSF) and data splits (V1 training and V1 validation); gene-level dependence plots were then generated for these drivers.

### 4.13 Quantitative real-time PCR

Total RNA was extracted from PBMCs using TRIzol reagent (Thermo Fisher Scientific). RNA was reverse-transcribed into cDNA using EVO M-MLV RT Premix (Agbio, AG11706). Quantitative real-time PCR (RT-qPCR) was performed on the ABI-7200 Real-Time PCR System (Applied Biosystems) using the SYBR Green Premix Pro Taq HS qPCR Kit (Agbio, AG11701). Primer sequences are listed in **Supplementary Table 14**.

### 4.14 Multiplex immunofluorescence staining

Pancreatic tissues from NOD and ICR mice were fixed in 4% paraformaldehyde for 24 h, dehydrated, paraffin-embedded, and sectioned at 4 μm. After antigen retrieval and blocking, sections were incubated with primary antibodies overnight at 4 °C. On the following day, corresponding HRP-conjugated secondary antibodies and TSA reagents were applied sequentially for multiplex labeling. Nuclei were counterstained with DAPI, and images were captured using a fluorescence microscope. Primary antibodies included anti-SOX4 (Bioss, bs-11208R, 1:1000), anti-PID1 (Bioss, bs-8065R, 1:1000), anti-PHLDA1 (Bioss, bs-1144R, 1:1000), anti-DUSP2 (Bioss, bs-7609R, 1:1000), anti-CD68 (Servicebio, GB153109, 1:5000), and anti-Insulin (Servicebio, GB12335, 1:10000).

### 4.15 Statistical analysis

All statistical analyses were performed using R software (version 4.2.0). Continuous variables are presented as mean ± standard deviation (SD) or median [Q1, Q3], depending on their distribution. Categorical variables are expressed as proportions. For normally distributed variables, comparisons between two groups were performed using the independent-samples t test, whereas comparisons among multiple groups were conducted using one-way analysis of variance (ANOVA). For non-normally distributed variables, comparisons between two groups were performed using the Mann–Whitney U test, whereas comparisons among multiple groups were conducted using the Kruskal–Wallis test. For ANOVA-based multi-group analyses, Tukey’s *post-hoc* test was used for pairwise comparisons. Comparisons of proportions were carried out using the chi-squared (χ²) test or Fisher’s exact test when appropriate. Pearson’s correlation coefficients were calculated to evaluate relationships between continuous variables. Mantel tests were conducted to assess relationships between PMRG-related expression patterns and immune score matrices. For high-dimensional analyses, including bulk transcriptome-wide differential expression, pseudotime-associated differential expression, and GO/KEGG/GSEA enrichment analyses, multiple testing was controlled using the Benjamini–Hochberg false discovery rate procedure unless otherwise specified. For single-cell subgroup marker analyses, adjusted P values reported by the Seurat FindAllMarkers function were used. Adjusted P < 0.05 was considered statistically significant for analyses with multiple-testing correction; otherwise, nominal P < 0.05 was considered statistically significant.

## Data Availability

The processed expression matrix for the 13-case in-house PBMC cohort generated in this study have been deposited in figshare and are available via the following persistent DOI: https://doi.org/10.6084/m9.figshare.32288148. The other datasets presented in this study can be found in online repositories. The names of the repository/repositories and accession number(s) can be found in the article/**Supplementary Material**.
