# Supplementary material for: From islet to blood: macrophage remodeling signatures for diagnosis and risk stratification in type 1 diabetes
Source: Front Immunol. 2026 Jun 26;17:1832672. doi: 10.3389/fimmu.2026.1832672 (PMC13350049; doi:10.3389/fimmu.2026.1832672)
Supplement: Supplementary file 1 [file Supplementaryfile1.pdf]

Supplementary Figures

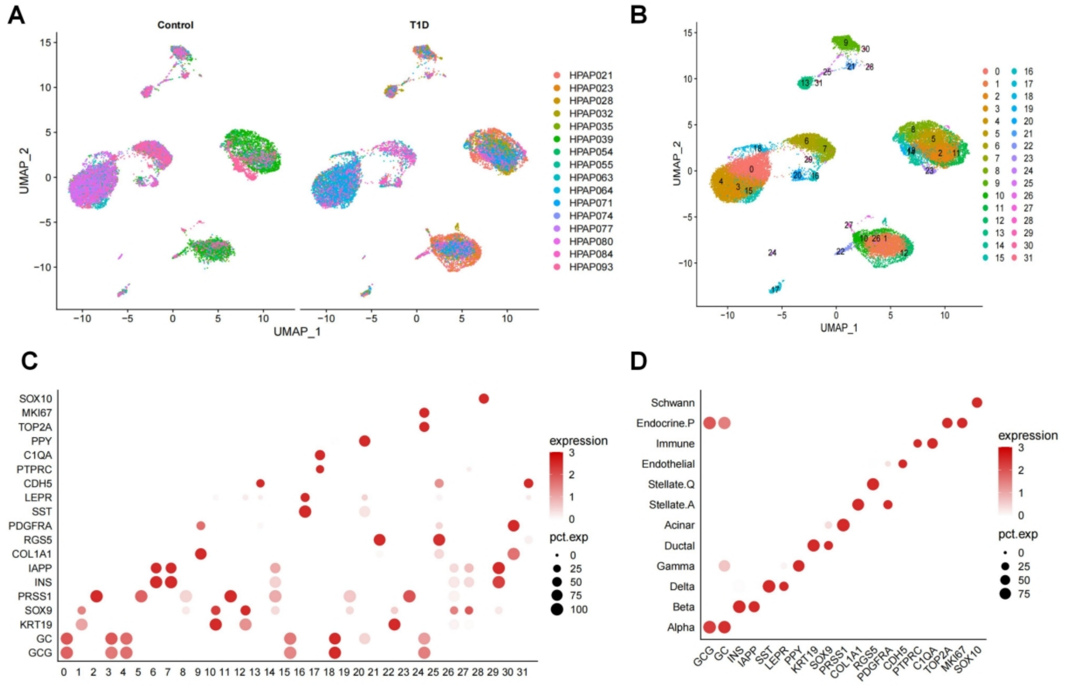

Supplementary Figure 1. Cellular Atlas of the Islet Microenvironment.

(A) UMAP visualization of scRNA-seq data after batch correction.

(B) UMAP dimensionality reduction clustering was performed on islet cells, resulting in 32 cell clusters.

(C) The FindAllMarkers function was employed to screen differentially expressed genes among the various cell clusters, visualized using bubble plots.

(D) Bubble plots visualized the marker genes of each cell type.

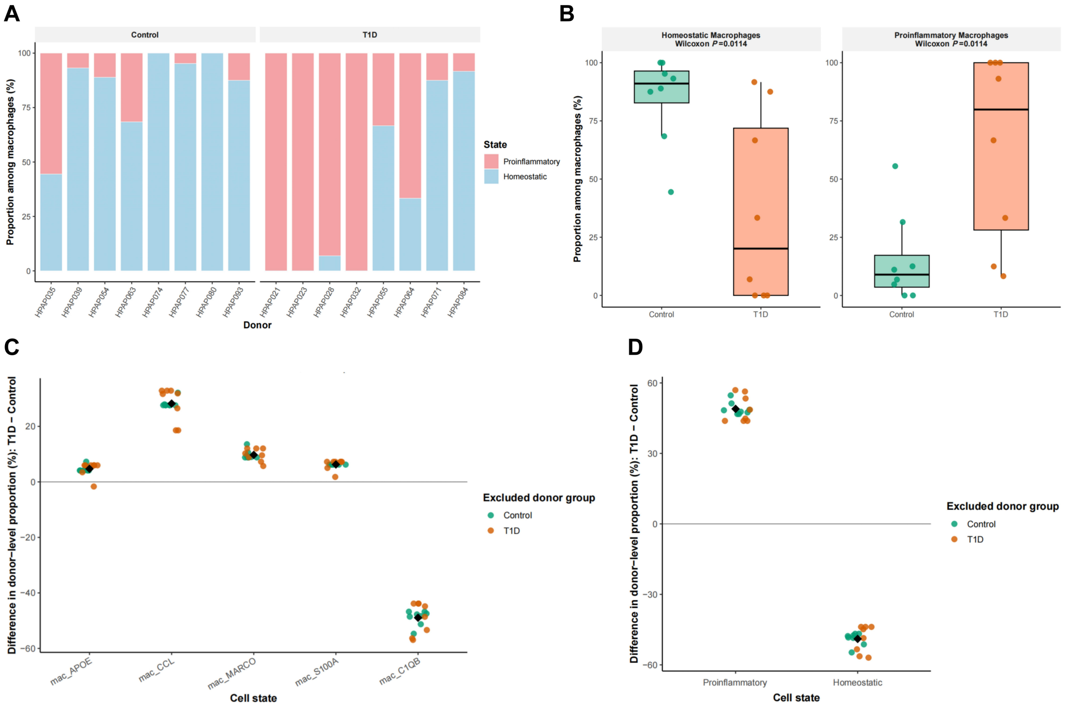

Supplementary Figure 2. Donor-level validation of the compositional shift toward proinflammatory macrophages in T1D.

(A) Donor-level proportions of proinflammatory and homeostatic macrophage states in control and T1D samples.

(B) Comparison of group-level proportions of proinflammatory and homeostatic macrophage states between control and T1D samples.

(C) Leave-one-donor-out analysis of the proportion of proinflammatory macrophages.

(D) Leave-one-donor-out analysis of the proportion of homeostatic macrophages.

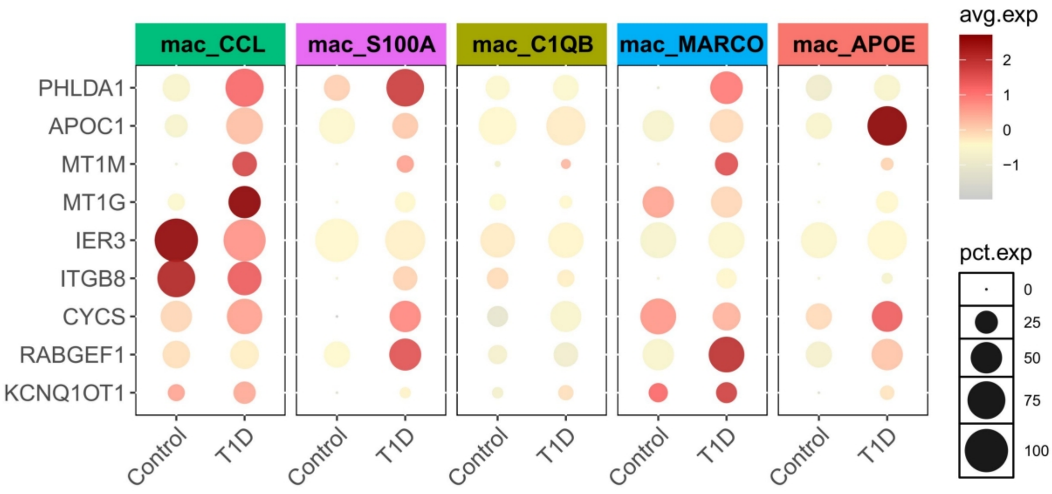

Supplementary Figure 3. DotPlot of nine iPMRGs expression across subgroups and conditions.

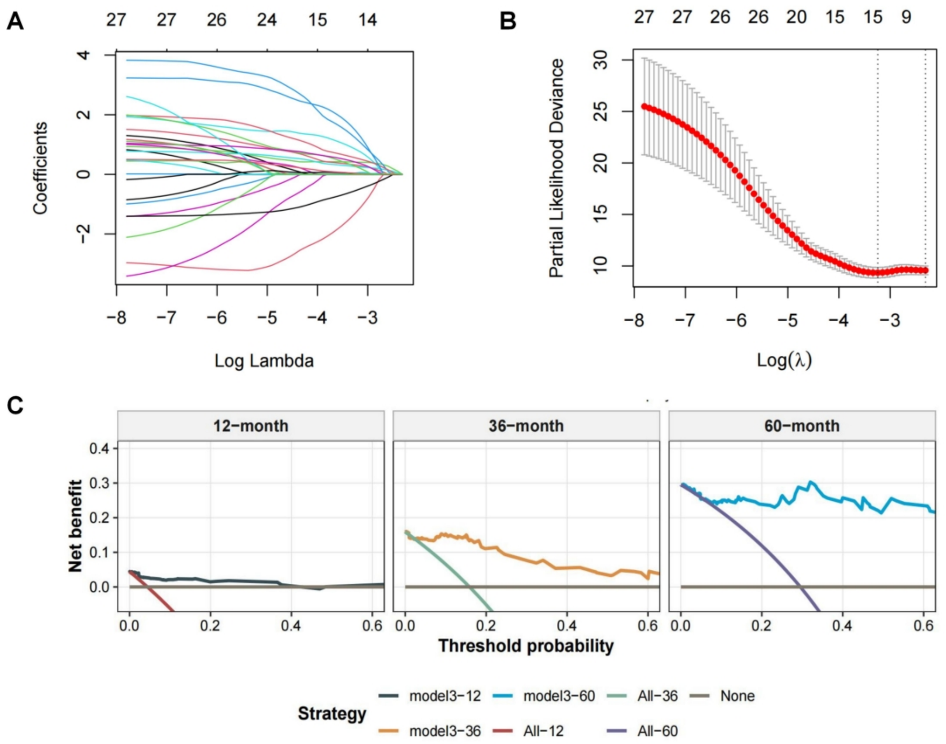

Supplementary Figure 4. LASSO Cox feature reduction and decision curve analysis of the PMRG-based prediction model.

(A) Coefficient profiles of candidate prognostic PMRGs in the LASSO Cox model.

(B) Ten-fold cross-validation for selecting the penalty parameter. The minimum-deviance criterion identified a fixed 15-PMRG panel, which was used for PMRG risk-score construction and downstream repeated-CV model evaluation.

(C) Decision curve analysis at 12, 36, and 60 months showing net benefit across threshold probabilities for the integrated Clinical-HLA-PMRG model.

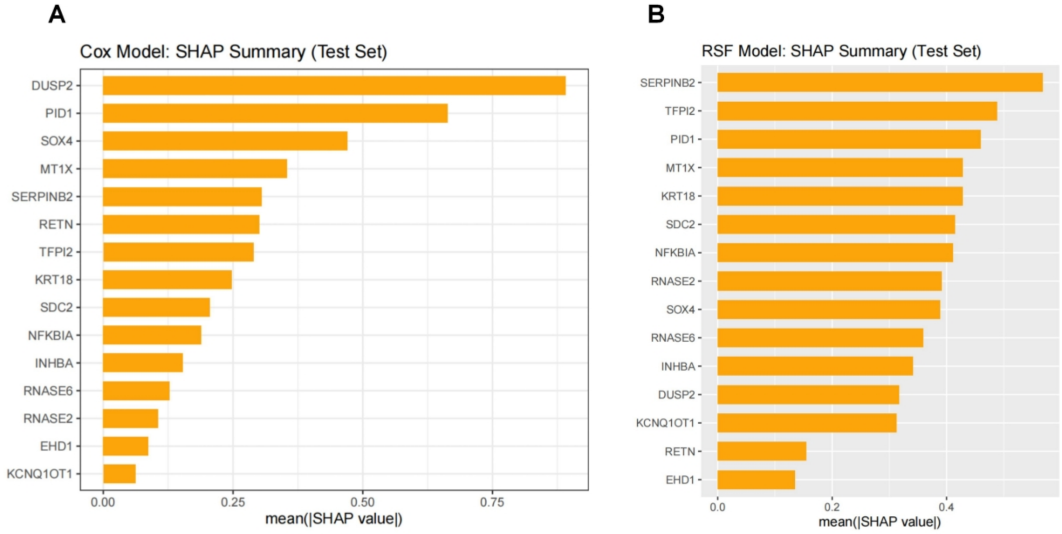

Supplementary Figure 5. SHAP-based comparison of PMRG drivers in Cox and RSF models.

(A) SHAP summary plot of individual prognostic PMRGs in the gene-level Cox model using testing subset.

(B) SHAP summary plot of individual prognostic PMRGs in the gene-level RSF model using testing subset.

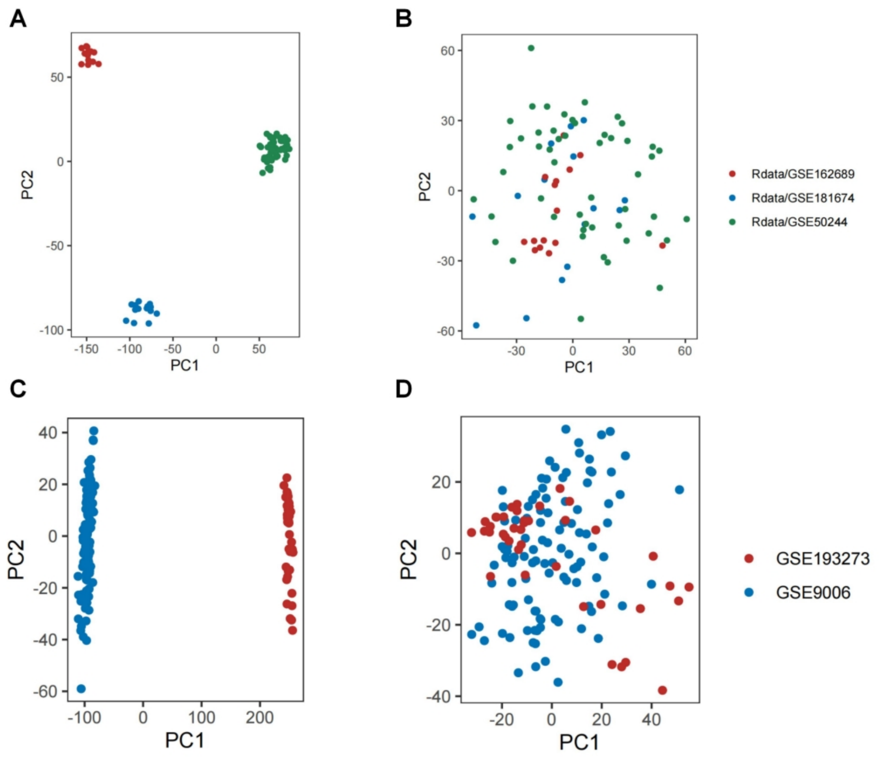

Supplementary Figure 6. PCA assessment of batch correction in integrated transcriptomic datasets.

(A)(B) PCA plots of integrated islet bulk RNA-seq datasets before and after batch correction.

(C)(D) PCA plots of integrated PBMC transcriptomic datasets before and after batch correction.
